# When tainted money should fund public goods: fundraising professional and public moral preferences

**DOI:** 10.1093/pnasnexus/pgad285

**Published:** 2023-09-26

**Authors:** Zoe Rahwan, Christina Leuker

**Affiliations:** Center for Adaptive Rationality, Max Planck Institute for Human Development, Lentzeallee 94, 14195, Berlin, Germany; Center for Adaptive Rationality, Max Planck Institute for Human Development, Lentzeallee 94, 14195, Berlin, Germany

**Keywords:** moral decision-making, moral preferences, tainted money, philanthropy, moral emotions

## Abstract

Philanthropy is essential to public goods such as education and research, arts and culture, and the provision of services to those in need. Providers of public goods commonly struggle with the dilemma of whether to accept donations from morally tainted donors. Ethicists also disagree on how to manage tainted donations. Forgoing such donations reduces opportunities for societal well-being and advancement; however, accepting them can damage institutional and individual reputations. Half of professional fundraisers have faced tainted donors, but only around a third of their institutions had relevant policies (*n* = 52). Here, we draw on two large samples of US laypeople (*n*s = 2,019; 2,566) and a unique sample of experts (professional fundraisers, *n* = 694) to provide empirical insights into various aspects of tainted donations that affect moral acceptability: the nature of the moral taint (criminal or morally ambiguous behavior), donation size, anonymity, and institution type. We find interesting patterns of convergence (rejecting criminal donations), divergence (professionals’ aversion to large tainted donations), and indifference (marginal role of anonymity) across the samples. Laypeople also applied slightly higher standards to universities and museums than to charities. Our results provide evidence of how complex moral trade-offs are resolved differentially, and can thus motivate and inform policy development for institutions dealing with controversial donors.

**Significance Statement**

Education, research, cultural, and charitable institutions rely on philanthropy to promote societal well-being and advancement. The risks of accepting morally tainted donations—a prospect which half of fundraisers face—may eclipse the material benefits. We tested whether key features of tainted donations—nature of the moral taint (criminal vs. morally ambiguous donors), donation size, anonymity, type of recipient institution—affect acceptability in large samples of US laypeople and professional fundraisers. Professional fundraisers were generally more conservative than laypeople in accepting donations and also more likely to reject large donations. Anonymity only marginally affects acceptability. These findings extend the tainted money literature to charitable giving and can be used to inform policies on controversial donors—which do not exist in the majority of organizations—and motivate new questions in applied ethics.

## Introduction

In the United States, charitable giving alone recently approached US$485 billion per year (1). Science philanthropy, including endowment income, constitutes nearly 30% of annual research funds at 50 leading US universities (2). Facing both public funding pressures and an upsurge of individual and foreign state donors, institutions are regularly confronted with the dilemma of whether to accept morally tainted donations—funds associated with criminal or morally ambiguous behavior—but often lack adequate guidelines for resolving these dilemmas (3–6). Indeed, we find that half of fundraising professionals (*n* = 52) have exposure to tainted donors, and almost two-thirds indicated a lack of direct policy guidance on how to address such situations. On the one hand, donations mean more opportunities for societal well-being and advancement. On the other hand, accepting controversial donations can cause public outrage and long-term reputational damage (7–9).

Opinions on how to resolve this dilemma differ (10, 11), including among professional ethicists (12, 13). In this study, we respond to the call for more research (14)—particularly *use-inspired* research (15)—in order to advance the moral psychology literature and provide concrete policy recommendations for practitioners. We conducted experiments with US laypeople and professional fundraisers to understand which key aspects influence the acceptability of controversial donations—donor type, donation size, anonymity, and institution type—their relative importance, and the role of moral emotions.

We found that donor type plays the most important role, with the public and professionals broadly in agreement on rejecting donations from criminals and accepting them from morally ambiguous individuals (though not foreign governments). The groups diverged on donation size, with professionals more hesitant to accept large donations from either criminal or morally ambiguous donors, indicating an absence of efficiency–morality trade-offs. Anonymity played only a minor role in increasing the acceptability of tainted donations for both samples. Finally, the public applied a higher moral standard to universities and museums than to human services nonprofits, often referred to as “charities.” As expected, moral emotions of anger and disgust were correlated with acceptability of tainted donations.

### Fewer cookies from an evil puppet

Money is not truly fungible. People have a deep-seated aversion to money associated with wrongdoing (16–18). This aversion has deep roots: even 1-y-olds prefer one cookie from a “good” puppet over two cookies from an “evil” puppet (19). When the offer of money—or cookies—is large enough, however, people can overcome their moral aversion (16, 19). Similarly, institutions offered a large donation from a morally tainted donor must engage in difficult efficiency–morality trade-offs (20).

Historically, the United States, and by extension the world, has benefited enormously from donations to education, research, and the arts from people commonly viewed as “robber barons” (e.g. John D. Rockefeller, Andrew Carnegie, and people with publicly declared anti-Semitic comments, e.g. Ford [21]). Foundations established by these individuals continue to this day to play a significant philanthropic role. More recently, universities, hospitals, and museums around the world have accepted funding from those convicted of a child sex crime (e.g. Jeffrey Epstein [5, 22]), trusts established by individuals with controversial ideologies (e.g. the Mosely family [23]), donors whose moral character has been questioned (e.g. the Sackler family [24], the Zuckerberg family [25, 26]), and regimes considered by some western countries to be authoritarian or otherwise problematic (e.g. Libya [4], Saudi Arabia [27], China [28, 29], and Russia [30]). In many of these cases, the recipient institution was aware of these moral taints before a donor relationship was established (e.g. in the education sector alone, between Epstein and Massachusetts Institute of Technology (MIT) [5], the Libyan Government and the London School of Economics and Political Science (LSE) [4]). The Vice Chancellor of Oxford University highlighted the dilemma in his comments regarding a record donation from controversial US financier Stephen Schwarzman: “Do you really think we should turn down the biggest gift in modern times, which will enable hundreds of academics, thousands of students to do cutting-edge work in the humanities?” (31).

Like the Oxford University Vice Chancellor, one can argue that a morally tainted individual's fortune is in better hands at institutions that produce public goods. At the same time, morally tainted donors may bring unwanted attention, complicity (32), or influence (33) to an institution and can jeopardize relationships with existing and future donors. Accepting tainted donations can also put staff members in uncomfortable or dangerous situations (5, 22) and alienate the public (34–36). There may also be indirect consequences: turning a blind eye to criminal or morally questionable donors (37) may encourage other institutions to do the same (5) and implicitly license further undesirable behavior (38, 39).

So what are institutions to do when facing controversial donations? To begin to answer that question, we sought to determine which aspects of tainted donations may affect their perceived acceptability, drawing on previous work in the field of moral psychology, guidance from fundraising professionals, and inspiration from Lawrence Lessig's reflections on MIT's Epstein donation scandal (12). Briefly, Lessig—a leading legal scholar—distinguishes the type of donor and the source of their funds (whether or not they derive from harmful or immoral behavior); he suggests that if donations derived from harmful or immoral behavior are to be accepted (as distinct from those deriving their wealth from morally ambiguous sources), it should be done so anonymously to prevent reputation white-washing. The size of the donation is important for understanding if and when people engage in efficiency–morality trade-offs (20); varying the institution type receiving donations makes it possible to gauge the generalizability of results. In the following, we consider each of these four factors in turn.

### What aspects of controversial donations matter?

The first aspect we examined was the type of donor (Table 1). Inspired by Lessig (12), we were intrigued to learn whether people distinguished between three different types of donor: those who had done “nothing but good” like Tom Hanks, those associated with morally ambiguous behavior (e.g. running a company that some consider a force for good and transformation in society and others see as dangerous; Lessig gives the examples of Google and Facebook), and convicted criminals. We anticipated that the acceptability of donations would decline in order of these donor types. To assess the generalizability of findings, we varied both the source of moral ambiguity (i.e. individuals associated with racism, data privacy violations, and environmental violations) and the type of criminal conviction (white-collar—health and investment frauds, and violent crime—sexual assault). The types of criminal convictions were motivated by events relating to the Sackler family (owners of Purdue Pharma—widely attributed to have contributed to the opioid crisis in the United States), Bernie Madoff and Jeffrey Epstein, respectively.

**Table 1.** Donor types.

Study 1	
Condition	Extract from vignette
1. “nothing but good”	The potential donor has no criminal convictions. The funds proposed for the donation were generated from doing nothing but good.
2. Criminal—sexual assault. Funds not from crime	The potential donor has been convicted of sexual assault. Specifically, the potential donor sexually abused women on multiple occasions. The funds proposed for the donation have not been generated from the crimes.
3. Criminal—white-collar (investment fraud) Funds not from crime	The potential donor has been convicted of a white-collar crime. Specifically, the potential donor created fraudulent investment schemes. The funds proposed for the donation have not been generated from the crimes.
4. Criminal—white-collar (investment fraud) Funds from crime	The potential donor has been convicted of a white-collar crime. Specifically, the potential donor created fraudulent investment schemes. The funds proposed for the donation have been generated from the crime.
5. Criminal—white-collar (health fraud) Funds from crime	The potential donor has been convicted of a white-collar crime. Specifically, the potential donor has not disclosed severe health risks associated with their best-selling product. The funds proposed for the donation have been generated from the crime.
**Studies 2 and 3**	
6. Criminal—white-collar (investment fraud)	The potential donor was a CEO at a large company. The donor was convicted of a white-collar crime while working at the company. Specifically, the potential donor helped to create fraudulent investment schemes.
7. Morally ambiguous—consumer data privacy	The potential donor is a CEO from a large company. The company produces goods and services that are considered by some to change society for the better. For others, the activities of the company raise significant concerns regarding consumer data privacy. The company has no recent convictions.
8. Morally ambiguous—environment	The potential donor is a CEO at a large company. The company produces goods and services that are considered by some to change society for the better. For others, the activities of the company raise significant concerns regarding environmental practices. The company has no recent convictions.
9. Morally ambiguous—racism	The potential donor is a CEO at a large company. The company produces goods and services that are considered by some to change society for the better. For others, the activities of the company raise significant concerns regarding racist practices. The company has no recent convictions.

The source of funds is ambiguous for all donor types in studies 2 and 3.

Prior research shows that people are more concerned by funds being generated in a tainted manner than by the association with a morally tainted individual and that the combination of a tainted person and a tainted source of funds is the least appealing (16). There may be situations which contradict this ordering, for example, if the individual is severely tainted (e.g. Epstein). We therefore also varied, among criminals, whether the funds were generated from the crime or not. The external validity of these scenarios is limited, as laypeople typically lack precise knowledge of donation sources and the possibility of donor funds originating from a crime is constrained by the common practice of governments seizing criminal proceeds. Still, in keeping with Tasimi and Gelman (16), we expected that among tainted criminal donors, donations sourced from tainted means would be less desirable than those from untainted means. While the government has powers to seize proceeds from crimes, this remains a relevant consideration for donors closely associated with convicted criminals (e.g. family members, associates)—their donations could reasonably be construed as being derived from criminal activity.

The second aspect we examined was the anonymity of the donation—specifically, whether or not the name and donation amount would be made public. Anonymity has been proposed as a means of allowing institutions to benefit from tainted donations while minimizing any institutional reputational damage (40) and avoiding other adverse effects, such as white-washing a donor's reputation (5, 12). In other words, anonymity has been considered a means to avoid any loss of morality while retaining efficiency, thereby resolving the trade-off. However, anonymity implies a lack of transparency (10, 13), and can paradoxically boost the reputation of the donor if the donation becomes known anyhow (41). In terms of second-round effects, institutions insisting on the anonymity of tainted donations may face psychological reactance from stakeholders unhappy with the attempt to suppress this information, as was the case for MIT with the Epstein donation (5). Nevertheless, we expected to observe an overall preference for anonymity.

The third aspect we examined was the size of the donation. As mentioned above, a large donation may generate sufficient good for an institution to overlook moral concerns (16, 19, 20). Accepting a morally tainted donation can elicit repugnance, but if the benefits outweigh the costs, preferences against the transaction can be reversed, resulting in an efficiency–morality trade-off (20). In contrast, even a small donation may be enough to generate disproportionate reputational and other costs for an institution, especially given the considerable role of social media in spreading information, especially that laden with strong negative emotions (7). Accordingly, we did not formulate a directional hypothesis on the relationship between donation size and acceptability.

The fourth aspect we explored was how the type of recipient institution affected the donations’ perceived moral acceptability. In particular, we were interested to understand if there was heterogeneity in the moral standards asserted by scientific institutions, cultural institutions like museums, and human services charities. In the absence of relevant empirical evidence, our a priori expectation was that the acceptability of tainted donations would be largely unaffected by the nature of the recipient institution (i.e. university, museum, or charity). Still, one could argue that charities differ from other institutions by the absence of alternative revenue streams prompting greater tolerance for receiving tainted donations.

Finally, given the increased role of foreign state philanthropy, notably in science (27, 42–44), we ran an exploratory scenario involving a morally ambiguous state actor involved in either human rights violations or environmental violations. We expected a greater aversion to such donations relative to equivalent donations from tainted individuals.

### The importance of understanding the moral preferences of both laypeople and fund-raising professionals

The moral preferences of both laypeople and experts toward charitable donations—especially where they affect public goods—are valid concerns, as both can inform the debate (45). Indeed, the very nature of dilemmas means that there may be various legitimate ways to resolve them. Further, fundraising professionals and members of the general public have different incentives for responding to the dilemmas: Professional fundraisers have a greater incentive to prioritize an institution's immediate financial requirements, whereas members of the general public may be more motivated to signal moral outrage, notably on social media. Professionals may thus be more likely than the public to accept tainted donations, and large ones at that. It was an open question if or how anonymity, donor type, and institution type would differentially affect the two groups’ response to the dilemmas. Any differences in preferences between the public and professionals may indicate opportunities for or risks to prevailing policies on controversial donors. If professionals hold more conservative preferences than the public, for instance, they may be unnecessarily foregoing tainted donations, diminishing the potential for public good provision. Alternatively, if professionals are more tolerant of tainted donations than the public, they may be taking on excessive risks to their institution's reputation.

To our knowledge, this is the first examination of these aspects of tainted donations among dedicated fundraising professionals and the first instance of contrasting the findings with results from laypeople, which provides unique empirical insights that can inform policy recommendations. In addition, our work enables an assessment of whether moral intuitions of prominent intellectuals—which themselves often lead to conflicting recommendations (12, 13)—are shared by these groups. More broadly, our work expands the study of tainted money to the domain of charitable giving, and motivates new questions within the field of applied ethics.

### Experimental approach

We report the findings from three preregistered online studies conducted between December 2019 and March 2021 (studies 1–3), and a preregistered follow-up study conducted in March 2023 to test for possible confounds (study 4), and a survey of professional fundraisers conducted in March 2023 (study 5).

All studies were programmed on Qualtrics survey software. Participants in studies 1, 2, and 4, which assessed the preferences of laypeople (*n*s = 2,019; 2,566; 600), were recruited from the Prolific platform. Although the platform was not able to provide a representative sample of the size needed to provide adequate power, this shortcoming does not appear to have impacted our main findings (see Methods), consistent with other findings (46).

Participants in study 3 (*n* = 694), which assessed the preferences of fundraising professionals, were drawn from US members of the Association of Fundraising Professionals (AFPs). The AFP is the only professional fundraising body in the United States with an enforceable ethics code. Its standards and principles, while relevant for managing controversial donations, do not provide specific guidance on such donations. Its US membership is collectively responsible for raising over USD100 billion annually (47)—more than one-fifth of total US annual fundraising (1).

We designed vignettes to examine how key aspects of tainted donations—donor type, anonymity, donation size, and institution type—affected their perceived acceptability. Our design was predominantly *between-subjects*, with each participant presented a single vignette, for which the aspects of the donation decision had been randomly assigned. They were then asked, “What should [the/your] institution do?” Responses were given on a six-point scale (*definitely reject* to *definitely accept*), collapsible into a binary (*accept*/*reject* scale). In studies 2 and 3, we also asked how changes in the size and anonymity of the donation would affect the acceptability of the donation presented in the single vignette (*less acceptable*/*equally acceptable*/*more acceptable*). This addition made it possible to run *within-subject* analyses and assess their convergence with the *between-subject* findings.

The vignettes did not refer to known donors in order to future-proof results, minimize social desirability bias (48), and avoid variation in knowledge of and reactions to historical scandals. We also avoided overt references to the consequences of rejecting funds (e.g. reduced research funding), as a loss framing would be expected to buoy the acceptability of funds (49) and is typically absent from the public dialogue (32, 33, 35).

In study 1, our primary factors of interest were donor type (with a focus on different criminal behaviors) and anonymity, giving a 5 (donor type) × 2 (public/anonymous donation) design (see Table 1 for donor types). The donor types were inspired by the real-world cases of Jeffrey Epstein, the Sacklers, and Bernie Madoff, and represent a modest attempt to understand generalizability of findings (i.e. whether health- and investment-based frauds or violent and white-collar crimes were assessed differently). Further, we assessed how acceptability varied whether the funds were generated from a crime or not, building upon previous research (16) in a philanthropic context.

In study 2, we considered donor type (with a focus on different types of morally ambiguous donors), anonymity, and—distinct from study 1—donation size (large: US$100,000/small: US$100), giving a 4 × 2 × 2 design. We assessed three types of moral ambiguity: violations of environmental or data privacy practices and racism, again as a modest attempt at assessing generalizability of findings. The selected domains were driven by high-profile issues in the United States (e.g. climate change, criticism of data privacy practices in Big Tech, and the increased profile of the Black Lives Matter movement).

Study 3 largely mirrored study 2, with two exceptions: first, participants were fundraising professionals who were asked to consider their own institution when assessing donation acceptability rather than being randomly assigned an institution type—for which we did not anticipate significant variation. Second, we elicited normative expectations of what their professional peers would do in the same situation. This measure was incentivized for accuracy (50).

A follow-up study, study 4 (*n* = 600), was conducted to assess the presence of possible confounds created by variations in social information and attributions of individual responsibility found across criminal and morally ambiguous donors. We also conducted a survey, study 5 (*n* = 52), with AFP members to better understand the frequency and nature of controversial donors, and policies for dealing with them.

The experimental designs were reviewed and approved by the Internal Review Board of the Max Planck Institute for Human Development. For further details on design and analysis approach and preregistrations are available in Methods section.

## Results

Our research provides a barometer for gauging which aspects of donations—donor type, donation size, anonymity, and type of recipient institution—are likely to spark moral outrage and which are likely to be met with ambivalence or even approval among the public and professionals in the United States. Descriptive statistics for study 1 (*n* = 2,019), study 2 (*n* = 2,566), study 3 (*n* = 694), and study 4 (*n* = 600) are presented in Methods section, along with analyses of demographic gender representativeness for studies 1 and 2. Study 5 (*n* = 52) provides unique insights from fundraising professionals into the frequency and nature of tainted donors, as well as into policies regarding controversial donors.

### Expert experience with tainted donations and policies

Half of fundraising professionals surveyed revealed they had exposure to tainted donors (*M* = 0.50, CI_95%_ = [0.36, 0.64]), among both existing and prospective donors. The nature of controversial donors varied, with the bulk (*M* = 0.60, CI_95%_ = [0.41, 0.77]) having generated negative publicity due to their behavior or attitude, but not necessarily any civil or criminal legal action. Among fundraising professionals with direct exposure to tainted donors, the frequency of tainted donations was relatively low, with a median of 1 (range: 0.1 to 5) out of 100 donors with whom they interacted. One fundraising professional noted that a low frequency can belie the problem as even one tainted donor can cause irreparable damage to an institution, not unlike the use of a single nuclear weapon. Fundraising professionals commonly share the perception (*M* = 0.54, CI_95%_ = [0.39, 0.68]) that the frequency of such donors had risen over the past 5 years; this perception was even stronger among laypeople (*M* = 0.66, CI_95%_ = [0.62, 0.70]).

A minority of fundraising professionals reported that their institution had a policy for handling tainted donations in their organization (*M* = 0.35, CI_95%_ = [0.22, 0.49]). Of the 18 instances where such a policy did exist, only 4 forbade the acceptance of donations from criminals (*M* = 0.22, CI_95%_ = [0.06, 0.48]). Some of the professionals stated that tainted donors were handled on a case-by-case basis, rather than according to a blanket policy on tainted donors. Laypeople showed only a weak preference for a policy that forbade criminal donations (*M* = 0.33, CI_95%_ = [0.29, 0.37]).

### Donor type

**Fig. 1.**
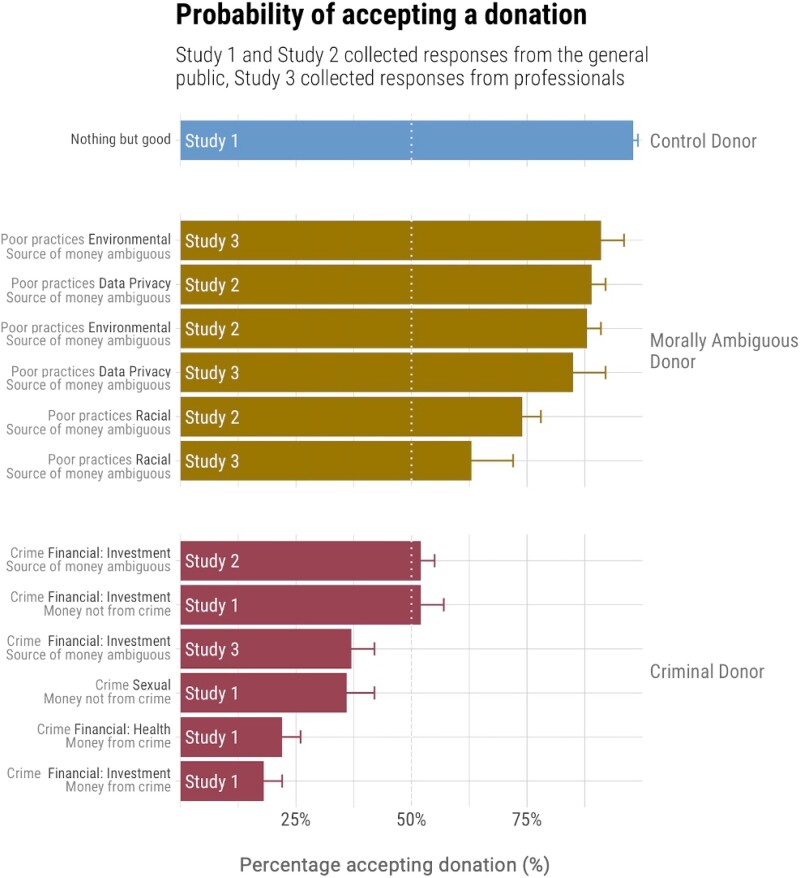
Donation acceptability by donor type. In studies 1 (*n* = 2,019) and 2 (*n* = 2,566), the public was generally against accepting donations from criminals, but tolerant, on average, of accepting donations from morally ambiguous donors. In study 3 (*n* = 694), fundraising professionals’ preferences were similar to those of the general public, but they were generally less likely to accept tainted donations and on average rejected donations from white-collar criminals. Data are means and 95% CIs.

We compared two broad types of moral taint: outright criminality and morally ambiguous behavior (see Fig. 1 and Table S1). We first address violent and white-collar crimes and whether or not the donated funds were generated from the crime (i.e. fraudulent investment schemes). The public clearly rejected donations from people convicted of the violent crime of sexual assault (*M*_accept(study 1)_ = 0.36, CI_95%_ = [0.31, 0.41]) but held mixed views on accepting donations from people involved in the white-collar crime of investment fraud when the funds were not derived from the crime (*M*_accept(study 1)_ = 0.52, CI_95%_ = [0.47, 0.57]) or when the source of the donation was ambiguous (*M*_accept(study 2)_ = 0.52, CI_95%_ = [0.49, 0.55]; see Figs. S1 and S2 for distributions). Professional fundraisers, however, on average rejected donations from white-collar criminals when the source of funds was ambiguous (*M*_accept(study 3)_ = 0.37, CI_95%_ = [0.32, 0.42]).

As expected, ill-gotten gains were not considered acceptable. Laypeople mostly rejected funds generated from white-collar crime, whether investment fraud (*M*_accept(study1)_ = 0.18, CI_95%_ = [0.14, 0.22]) or health-related fraud (*M*_accept(study1)_ = 0.22, CI_95%_ = [0.18, 0.26]; see Table S1 and Fig. S1). In practice, this may be a moot point, as proceeds from crimes are usually seized, but it highlights the risk of accepting donations that may be perceived as connected to criminal or terrorist behavior (e.g. from relatives or associates of the perpetrators). This was recently borne out by recent revelations that the bin Laden family made a substantial donation to The Prince of Wales's Charitable Fund (51).

Next, we turn to morally ambiguous donors—those perceived by some as benetting society and by others as engaging in some form of moral, though not criminal, violation. Both laypeople and fundraising professionals on average said they would accept donations from morally ambiguous donors (*M*_accept(public)_ = 0.84, CI_95%_ = [0.82, 0.86], *M*_accept(professionals)_ = 0.80, CI_95%_ = [0.76, 0.84]), though professionals were slightly more cautious (*Z*[Wilcoxon rank-sum test]_one-sided_ = 240,500, *P* = 0.038). The greater tolerance for donations originating from morally ambiguous donors relative to criminal donors was in line with our expectations.

We ran a follow-up experiment, study 4, to ensure that these findings were not confounded by differences in the vignettes with respect to social information or individual responsibility. When adding social information (i.e. that the firm did “good” as well as caused harm) to a criminal donor vignette, and when emphasizing individual responsibility in generating moral ambiguity in a noncriminal vignette, we found no differences (*Z*_one-sided_ = 11,014, *P* = 0.312, *Z*_one-sided_ = 10,735, *P* = 0.858). Furthermore, when controlling for both social information and individual responsibility in addition to other aspects—institution type, donation size, and donation anonymity—participants still preferred morally ambiguous donations over criminal donations (*Z*_one-sided_ = 8,290.5, *P* < 0.001; see Fig. S9).

Considering morally ambiguous donors, both the public and professionals were less tolerant of racism (*M*_accept(public)_ = 0.74, CI_95%_ = [0.70, 0.78], *M*_accept(professionals)_ = 0.63, CI_95%_ = [0.54, 0.72]) than of data privacy or environmental violations (*M*s > 85% for the public and professionals; see Fig. 1, Tables S1 and S11). This may reflect concerns about systemic racial injustice in the United States, recently highlighted by the Black Lives Matter movement (52).

Laypeople and professional fundraisers both had lower tolerance for institutions receiving large tainted donations from foreign governments relative to equivalent donations from individuals (see Fig. S6). Further, among tainted donations from foreign governments, both groups expressed a greater aversion to donations associated with governments believed to be involved in human rights abuses than in environmental violations (see Table S5). Wariness of state-level philanthropy may be warranted as it is increasingly used as a foreign policy tool (42, 53), and has led to arrests and even criminal convictions for individual scientists who failed to disclose financial relationships with foreign entities (43, 54).

Generally, fundraising professionals were more averse than laypeople to accepting donations from either a white-collar criminal or a morally ambiguous donor (Table S11). Fundraising professionals’ expectations of their peers’ attitudes (incentivized for accuracy), did not meaningfully deviate from individual judgments of tainted donation acceptability (see Fig. S7). This offers convergent evidence that professionals are more conservative than laypeople. Rather, professionals’ greater reluctance to accept tainted donations may reflect a deeper understanding of long-term reputational damage and the personal consequences of accepting controversial donations, even though it also means lost fundraising opportunities in the near term.

### Donation size

**Fig. 2.**
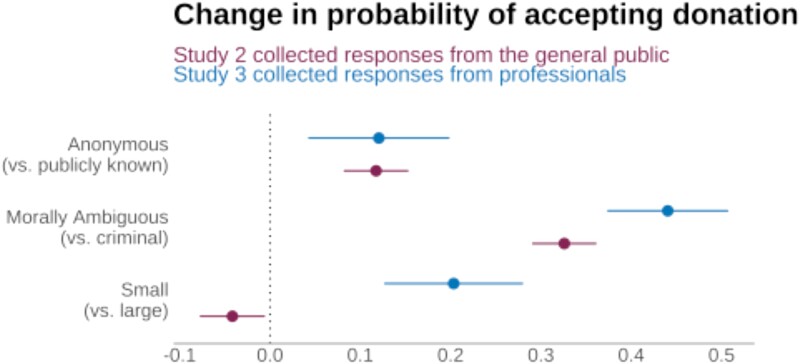
Donation acceptability by key aspects. Donor type had the strongest influence on donation acceptability with both the general public (study 2: *n* = 2,566) and fundraising professionals (study 3: *n* = 694) preferred that institutions accept donations from morally ambiguous donors over criminal donors. Donation size had an asymmetric effect among the groups, with professionals exhibiting a strong aversion to large donations from criminals. Both professionals and the public preferred anonymity for all morally tainted donors. Data are marginal effects with standard errors from a linear regression model with size, donor type, and anonymity as covariates. All estimates have *P* < 0.05.

Consistent with this apparent sensitivity to institutional—and perhaps personal—reputational risk, professional fundraisers were more averse to large (US$100,000—*M*_accept_ = 0.50, CI_95%_ = [0.44, 0.55]) than small (US$100—*M*_accept_ = 0.67, CI_95%_ = [0.62, 0.72]) donations from tainted donors (*Z*_one-sided_ = 70,469, *P* < 0.001; see Fig. 2 and Table S10). Large donations from tainted donors split the population participants (see bimodal distribution of donation acceptability in Fig. S5), though there was a particular aversion to large criminal donations (*M*_accept_ = 0.25, CI_95%_ = [0.19, 0.31]). In contrast, respondents from the general public had a weak preference for large donations (*Z*_one-sided_ = 790,287, *P* = 0.014), except when they were from criminals: here, large donations were unacceptable to roughly half of laypeople (*M*_accept_ = 0.49, CI_95%_ = [0.45, 0.53]; see Table S3). Both laypeople's weak preference for and professionals’ aversion to large donations were robust in the within-subject analyses (see Table S12).

### Donation anonymity

In line with our expectations, we found evidence that anonymity boosts the acceptability of tainted donations among both laypeople and professionals (*Z*_study1,one-sided_ = 456,790, *P* < 0.001; *Z*_study2,one-sided_ = 738,881, *P* < 0.001; *Z*_study3,one-sided_ = 54,160, *P* = 0.004), though the preference was fairly weak (Cohen's *d*_study1_ = −0.21; *d*_study2_ = −0.21; *d*_study 3_ = −0.20; see Fig. 2, Tables S2 and S8–S10). Indeed, the effect of anonymity for fundraising professionals was not robust when other experimental and demographic control variables were included in the regression model (see Table S10). The majority of laypeople accepted donations from white-collar criminals under condition of anonymity and if the funds were not generated from the crime (see Table S2). Within-subject analyses were consistent with these results, also revealing a preference for anonymity that was more prevalent among laypeople than professionals (see Table S12). Both populations tended to think it was possible to maintain anonymity (see Table S7). Note that in our scenarios, anonymity referred to both the donor and the donation amount; there was no indication of whether anonymity was requested by the donor or the institution was not stated.

### Type of recipient institution

Laypeople consistently held universities and museums to slightly higher standards than charities (see Fig. S8, Tables S8 and S9). Among laypeople, donations generated by criminal means were only deemed acceptable, on average, for charities (see Table S4—study 1). In contrast, the type of institution that fundraising professionals worked at had no detectable bearing on their donation preferences (see Table S10).

### Role of moral emotions

Studies 1–3 showed a consistent pattern of moral emotions and trust when considering morally tainted donations: The more strongly a participant felt that a donation should not be accepted, the more anger and disgust—both emotions associated with moral outrage (55)—they expressed. The associations were strong in study 1, which focused on criminal donors, and moderate in studies 2 and 3, which focused on morally ambiguous donors (anger: *r*_public-study1_[1,618] = −0.80; *r*_public-study2_[2,565] = −0.42; *r*_professionals-study3_[693] = −0.32; disgust: *r*_public-study1_[1,618] = −0.82, *P* < 0.001; *r*_public-study2_[2,565] = −0.41, *P* < 0.001; *r*_professionals-study3_[693] = −0.29, all *P*s < 0.001; see Table S6). Furthermore, laypeople expected that accepting morally objectionable donations would have a small-to-moderate effect on trust in the institution (*r*_public-study1_[1,618] = 0.32, *P* < 0.001; *r*_public-study2_[2,565] = 0.52, *P* < 0.001), whereas professionals were more sensitive to possible loss of trust (*r*_professionals-study3_[693] = 0.72, *P* < 0.001).

## Discussion and conclusion

### Implications for understanding moral decision-making

Our work complements much of the existing literature on tainted money. Consistent with Tasimi and Gelman (16), we found in the context of charitable giving that the combination of a tainted donor and a tainted source of funds makes for the least acceptable donations. Whether the funds were generated from a crime dominated the nature of the moral taint among criminal donors in driving moral preferences. However, the type of criminal donor was also important. When a donation from a criminal was not derived from the proceeds of crime, laypeople differentiated between those convicted of white-collar and violent crimes, accepting funds from the former at the margin but not the latter. More broadly, both laypeople and fundraising professionals tended to differentiate acceptability of donations depending on whether the donor had a criminal conviction or not—donations from individuals thought to be engaged in morally ambiguous behavior were acceptable while those from criminals were generally deemed unacceptable. That is, the presence or absence of a conviction was the dominant cue for donation acceptability.

Neither laypeople nor fundraising professionals appeared to engage in efficiency–morality trade-offs (20). In fact, fundraising professionals were more cautious about accepting large donations, whereas laypeople were broadly indifferent to donation size. This suggests that deontological considerations of donations are dominant for both laypeople and professionals when determining donation acceptability. This is in contrast to the findings of Taylor (56), who found that donation size is the dominant factor in driving donation acceptability for funds from noncriminal donors among nonprofit managers (of whom only 20% spent more than half of their working time on fundraising). Whether substantially larger donations would encourage laypeople to consider efficiency–morality trade-offs or reverse the preferences of fundraising professionals remains an open question.

Moral questions cannot be disentangled from emotions (57, 58). Certainly, our findings challenge the notion of consequentialist considerations in explaining moral decision-making in this context and are more consistent with “social intuitionist” models (59). Further, a common finding in charitable giving specifically is that the moral decisions of laypeople tend to be driven by moral emotions (59) rather than by utilitarian considerations (60). Consistent with such findings, we found evidence of anger and disgust being associated with decisions to reject donations from morally tainted individuals—especially criminals. These considerations may help explain why laypeople care relatively little about the size of a donation when considering overall donation acceptability.

Anonymity is known to shape economic behaviors in general (61) and charitable giving in particular (62). Broadly consistent with previous research (56), we found that anonymity only modestly boosted the acceptability of tainted donations. However, there were important threshold effects in particular scenarios. Specifically, in cases of donations from white-collar criminals where the source of funds was not explicitly a crime, laypeople on average accepted donations under condition of anonymity. In general, laypeople also had a slightly stronger preference for anonymity of tainted donations and stronger belief in the ability to maintain that anonymity (*Z*_one-sided_ = 827,050, *P* = 0.004, *d* = 0.16). Further, it is possible that laypeople believed that anonymity was requested by the institution because of the moral taint of the donor; in reality, however, this rarely occurs (5). Overall, the weak preference for anonymity of tainted donations exhibited by both laypeople and experts reveals that moral decision-making in this context is not exclusively deontological. Rather, some weight seems to be implicitly given to the “good” or outcomes that can be achieved with the funds (63), consistent with a weak form of an efficiency–morality trade-off.

### Implications for the development of policies on tainted donors

We found that only a minority of charitable organizations (35%) currently have policies in place regarding controversial donors. Our empirical findings offer valuable insights that can help guide institutions in establishing or refining policies. Acknowledging national and institutional cultural variation and that norms change over time, our work identifies several broad principles that can be applied when establishing or reviewing philanthropic relationships in order to better manage the risks and opportunities that come with controversial donors.

First, caution is advised when considering donations from criminals, especially those convicted of violent crime. In contrast, money from morally ambiguous donors is deemed acceptable by laypeople and fundraising professionals and thus seems to require less scrutiny, though greater caution is recommended for donors associated with moral violations related to race (at least in the US context). Extra due diligence is warranted for morally ambiguous foreign state actors, especially where human rights violations are concerned; some institutions have already introduced such measures (64). The recent war in Ukraine demonstrates the crystallization of such risks for institutions such as MIT, which abandoned a project with the Russian government (44) or Harvard, which received funds from a Russian oligarch (30, 65).

Second, extra due diligence is also recommended in determining the source of funds if the donor is—or is associated with—a white-collar criminal. The argument that dirty money can be repurposed to a good cause is perceived as uncompelling to laypeople. In reality, information on whether funds were generated by a crime is likely inaccessible to laypeople. Rather, laypeople may have a general sense of funding sources, given the donor is a public figure. Moreover, the possibility of donor funds being generated from a crime is constrained by the common practice of asset forfeiture in criminal cases. Still, the effectiveness of asset forfeiture varies across jurisdictions and the residual risk remains of accepting donations from those associated with criminals who may be reasonably construed to have generated the funds for a donation. As such, fundraising professionals may benefit from commercial databases such as those used in finance for “politically exposed persons,” which could extend to donors’ relatives, and professional and social associates (66).

Third, anonymity is no panacea for institutions wishing to gain material benefits while mitigating reputational and other risks. Indeed, in practice, it is highly unusual for institutions to request anonymity (5); such requests usually originate from donors themselves, who may wish to avoid unwanted attention (e.g. from other fundraisers and tax authorities). Anonymity appears to be a tool that only marginally boosts the acceptability of controversial donors in the eyes of the general public. If deployed, the transparency expected by the end-users of the funds within an institution needs to be taken into consideration, especially with regard to foreign donors (43). In addition, revelations that tainted donors enjoyed anonymity can ultimately exacerbate reputational damage and stakeholder outrage (e.g. Epstein's donations to MIT).

Fourth, although larger donations—especially from criminal donors—may warrant additional professional scrutiny, donation size does not appear to be of great importance to the general public. One interpretation of this finding is that professional respondents’ caution could be depriving institutions of notable material benefits and, consequently, society of important contributions. Relatedly, a suggestion to blind institutions to the size of a donation to debias institutional evaluations of acceptability may result in an increase, rather than the speculated decrease (37), in tainted donations.

Finally, while in practice it may be difficult to make direct comparisons, educational and research institutions would benefit from more conservative donation policies relative to charities, since the public holds them to a higher moral standard. The greater tolerance of tainted donations to charities may stem from potential constraints on their ability to generate revenue (e.g. they cannot collect tuition or entrance fees) or that charities may address more pressing needs in society. While the perceived differences across institutions are small, the increased marginal probability of provoking high-impact moral outrage makes this point worthy of consideration. Institutions could use methods similar to those we have deployed here to generate bespoke insights into their stakeholders’ values and to track those preferences over time.

### Limitations

Our work is focused on the United States due to the prevalence of and dependence on philanthropy in general and science philanthropy specifically. Given previous work on cross-cultural moral preferences and behavior (67, 68), it is plausible that our findings generalize to countries with similar cultures. However, even within the United States, charities and other institutions may not be held to the same standards; for instance, we found the public to be more tolerant of tainted donations to charities than to universities. Even within a charitable domain values may differ between institutions—for instance, institutions guided by a value of giving “second chances,” may be less likely to veto tainted donations (69).

We chose to rely on a stated preferences approach for these studies. While eliciting revealed preferences is often considered a superior methodology for understanding preferences, this is not always the case for subjective decisions (70) and may be impracticable for moral dilemmas (71). In our studies, eliciting revealed preferences would have required participants to decide whether or not to accept tainted money donations. Such an approach creates ethical and practical difficulties: We did not want to deceptively label our research funds as being from tainted sources or to raise funds from tainted donors. Furthermore, this type of decision is only relevant for professional fundraisers; laypeople may judge institutions’ fundraising decisions but do not make them themselves.

We followed best practice for stated preference measures, such as complementing between-subject measures with within-subject measures and using incentivized measures for professionals—in order to be able to assess the robustness of our findings. The between-subject and within-subject measures were consistent. Further, our incentivized measures of peer expectations for fundraising professionals were strongly aligned with their individual decisions (see Fig. S7). This can be interpreted as an indication that individual stated preferences were largely unbiased (e.g. by a willingness to appear virtuous to experimenters). However, a “false consensus effect” (72) wherein individuals incorrectly overgeneralize their view to their peers, cannot be ruled out.

## Conclusion

Moral judgments are commonly guided by intuitive, emotional responses. This contributes to a feature of moral dilemmas of their resolution not being obvious. Further, resolutions may differ between groups such as experts and laypeople, as well as across time and cultures. The emotional nature of moral decisions also means that they are not necessarily calibrated for optimizing the public good. Rather than taking a normative position, our research provides what we understand to be the first empirical evidence on which aspects of tainted donations matter to the public and to fundraising professionals.

Philanthropic relationships are complex and dynamic. Reputational risks posed by controversial donors can be mitigated by robust due diligence and understanding stakeholder values; however, ongoing risk assessments for existing donors and strategies to counteract potential reputational risk (e.g. revoking naming rights, returning funds) are also important. A more refined calibration of the benefits and risks from donations—both initially and throughout the philanthropic relationship—will ensure that society can continue, in a sustained manner, to benefit from the extraordinary contributions philanthropy makes possible.

## Materials and methods

### Materials

All data, codes, preregistrations and surveys are available at: https://osf.io/b36sf/? view_only=c556843959ad4eeebf5376207bfd24f7

### Methods

This article is based on three online studies conducted between December 2019 and March 2021 to investigate which factors shape the acceptability of morally tainted donations. A fourth, follow-up, study was run in March 2023, as was a fifth study that examined professional fundraisers’ real-world experiences with controversial donors and donation policies. All studies were programmed on Qualtrics survey software. Participants were recruited from the Prolific platform (US residents only, fluent in English, minimum approval rate of 80%). In studies 3 and 5, participants were recruited from members of the AFPs (US members only).

### Study 1: experimental design

In study 1, *n* = 2,019 participants completed a survey posted on Prolific for a flat payment of GBP0.80 (∼USD1.0) for an average of 6 min of their time (interquartile range [IQR] = 3–7 min) in December 2019. Inclusion criteria were being a US citizen, fluency in English (self-assessed), and a minimum approval rate of 80% in earlier studies completed on the platform. The survey was approved by the Max Planck Institute for Human Development. No participants were excluded from data analysis, as per the preregistration. The sample size was selected to be able to detect small-sized effects (*d* = 0.3) with conventional power (*β* = 0.80) when conducting a two-sided *t*-test, within our funding constraints.

The final sample consisted of 992 people identified as female, 951 identified as male, 6 participants identified as transgender, 18 who identified as “other,” 5 who preferred not to specify, and 47 who did not indicate gender; the average age was 33 years (range: 18–80 years, SD = 12.81). While we would have preferred a representative sample, Prolific did not offer this service for samples larger than *n* = 1,000 in the United States at the time due to constraints relating to the size of their participant pool (73). However, we examined (i) to what extent our sample was representative in terms of gender and age—variables available through Prolific—and (ii) how any deviations from demographic representativeness as reported by Prolific from the US Census Bureau affected our results. Refer to “Studies 1 and 2: demographic representativeness” section below.

After obtaining informed consent, we randomly assigned participants to one of 10 conditions in our 5 (donor type) × 2 (public/anonymous donation) between-subject design. In addition, we randomly varied the institution receiving the donation (university, museum, or charity), expecting to collapse this aspect. The donor types comprised those who had acquired wealth by doing “nothing but good,” a violent criminal (convicted of sexual assault), and three types of white-collar criminals: one convicted of a health-related fraud and two convicted of financial fraud. This design enabled us to test whether participants distinguished between (i) violent and white-collar criminals, (ii) donor funds sourced from the crime vs. independent of the crime, and (iii) different types of white-collar crime: financial and health-related frauds.

Participants were presented a simple vignette, decontextualized to avoid deliberately evoking real-world examples, because their knowledge of and response to these would be heterogeneous. Further, decontextualized scenarios can reduce social desirability bias (48). Here is an example of a vignette (in the condition in which a donation made to a university by a white-collar criminal, would be anonymous, and the funds were not from the crime):

Consider a university which has just been approached by a potential donor looking to make a substantial donation. These potential donor funds are crucial for pursuing the mission of the university. The potential donor has been convicted of a white collar crime. Specifically, the potential donor created fraudulent investment schemes. The funds proposed for the donation have not been generated from the crime. The name of the donor and the donation amount would not be disclosed to the public.

At the end of the vignette, participants were asked “Should the {institution} accept this donation?” Participants selected a response from a six-point Likert scale (*definitely reject*, *likely reject*, *rather reject*, *rather accept*, *likely accept*, and *definitely accept*), collapsible into a dichotomous scale (accept/reject). The time taken to respond was recorded.

We then asked participants how angry and disgusted they were (seven-point Likert scale anchored with *not at all* and *extremely*), as these are moral emotions strongly correlated with moral repugnance (55). We also measured perceptions of how public trust would be affected if the institution were to accept the donation.

Participants were then asked in an open-text format to give reasons for both accepting and rejecting the donation. The data were not intended for analyses but rather to provide an opportunity for participants, especially those who may have felt social judgment for their decision, to unburden themselves by providing reasons for their own decisions and thinking about how others with opposing views may justify their decisions.

We then asked a series of questions on aspects that we believe may have influenced responses to the donation dilemma. Participants in the violent or white-collar crime conditions were asked if they had been a victim of unwanted sexual advances or investment fraud, respectively (*yes*, *no*, and *prefer not to answer*), and which individuals or companies came to mind when thinking of those engaged in sexual abuse, creating fraudulent investment schemes, or failing to disclose severe health risks. All participants were asked whether they had ever been employed by the kind of institution presented in the condition and the recency of their last visit. We also sought beliefs on the ability to maintain the anonymity of a donation (seven-point scale anchored with *extremely easy* and *extremely difficult*), and asked about the recency of their latest donation to charity. Finally, basic demographic information was collected (gender identity, age, household income, education, political preference, and religiosity).

### Study 2: experimental design

Study 2 shifted the focus from criminal donors to donors who ran companies associated with morally ambiguous behavior—that is, companies we defined as “considered by some to change society for the better … and for others, to raise significant concerns” regarding one of three issues (consumer data privacy practices, environmental practices, and racism). We also moved away from “substantial” donations in study 1 to explore how small (USD100) vs. large (USD100,000) donations would affect moral preferences. In summary, we had a 4 (donor type) × 2 (donation size) × 2 (anonymity) design. No explicit references to the source of the funds were made (i.e. it was left ambiguous).

Participants completed a survey posted on Prolific for a flat payment of GBP0.80 (∼USD1.0) for an average of 6 min of their time (IQR = 4–10 min) in October 2020. Inclusion criteria were being a US citizen, fluency in English (self-assessed), and a minimum approval rate of 80% in earlier studies completed on the platform.

We sought a sample of *n* = 2,480 based on a conservative bootstrapped power analysis of the study 1 findings. We aimed to be able to detect small-sized effects (*d* = 0.30) using rank-sum tests with 80% power. Samples were drawn with replacement in 50-subject increments up to a sample size of 1,000. The simulated samples were used to test for the presence of anonymity, where only small-sized effects had been found (*d* ∼ 0.2). Given that we had three main dependent variables—donor type, donation size, and anonymity—we set alpha at 0.0167 (*α* = 0.05/3). We thus needed ∼260 participants per condition to detect any meaningful differences between donor types (criminal vs. morally ambiguous). Given the possibility that we would not be able to collapse the types of morally ambiguous practices (poor consumer data privacy, poor environmental practices, or racism), we boosted each of the four main cells pertaining to morally ambiguous individuals by 100.

The survey was approved by the Internal Review Board of Max Planck Institute for Human Development. No participants were excluded from data analysis, as per the preregistration. The final sample (*n* = 2,566) consisted of 1,304 participants identifying as female, 1,183 participants identifying as male, 40 participants who identified as nonbinary, 4 who identified as “other,” 4 who preferred not to specify, and 31 who did not indicate gender; the average age was 32 years (range: 18–84 years, SD = 11.6). Again, while we would have preferred a representative sample, Prolific did not offer this service for samples larger than *n* = 1,000 in the United States at the time.

After running the study, we learned that the scale for donation acceptability—our key dependent variable—was partially mislabeled in one condition. While five of the six text labels on the scale were correct, the label on the far right-hand side of the scale was erroneously labeled *definitely reject* rather than *definitely accept*. This mislabeling occurred in the condition relating to a financial criminal (“type 6” donor) proposing a small donation that would be anonymous.

We re-ran this condition of the study, targeting *n* = 300; the minimum required for a representative sample at the time on Prolific and slightly above the target sample size of the original condition (*n* = 260). The survey was launched on 2021 January 29,. After excluding participants who did not give consent and did not complete the survey, we have the following sample sizes: *n*_original_ = 262, *n*_re-run_ = 299. Visual examination of the acceptability distributions (Fig. S10) indicates that participants in the original condition seemed to prefer to answer with the accurately labeled “likely accept” (=5) over the mislabeled extreme option “definitely reject” (which was intended as “definitely accept”) (=6) and that the remainder of the distribution was unaffected. Using both parametric and nonparametric tests, no significant differences (*α* = 0.05) emerged between the original and re-run study data (*t*_two-sided_ = −1.069, *P*-value = 0.29; *M*_original_ = 3.75, *M*_re-run_ = 3.88), *Z*_two-sided_ = 35,772, *P* = 0.007). The analyses presented in the main article use data from the re-run survey, which corrected for the mislabeled scale in this condition of the original study.

After informed consent was obtained, participants were randomly assigned to 1 of 8 conditions in our 2 (donor type) × 2 (public/anonymous donation) × 2 (small/large donation) between-subject design. Similarly to study 1, we randomly varied the institution receiving the donation: university, museum, or “social services nonprofit.” The latter institution was amended from “charity” in study 1 as we were advised that this term may cause confusion in the United States, where “charity” can be construed as any number of types of nonprofit, including universities, museums, hospitals, and social services organizations. To gauge the level of possible confusion, we also asked participants in study 2 which organizations came to mind when thinking of a charity. Results showed that they broadly thought of social services entities (notably the Red Cross) rather than educational or cultural institutions. Having found small effects of the type of institution on the acceptability of donations in study 1, we again expected to collapse this aspect.

Similarly to study 1, participants were presented a simple vignette. Here is an example of a vignette (in the condition in which a large, public donation was offered to a university by an [senior, decision-making] individual working at a company thought to be engaging in poor environmental practices):

Consider a university which has just been approached by a potential donor looking to make a donation. The potential donor is a CEO at a large company. The company produces goods and services that are considered by some to change society for the better. For others, the activities of the company raise significant concerns regarding environmental practices. The company has no recent convictions. The potential donor is seeking to donate $100,000. This is considered by the university to be a relatively large donation. The donation would be made public (i.e. the donor's name and donation amount would appear on an annual donor list).

At the end of the vignette, participants were asked “What should the {institution} do regarding this potential donation?” Participants selected a response from a six-point Likert scale (*definitely reject*, *likely reject*, *rather reject*, *rather accept*, *likely accept*, and *definitely accept*), collapsible into a dichotomous scale (*accept*/*reject*). The time taken to respond was recorded.

As in study 1, we then asked participants to give reasons for both accepting and rejecting the donation in an open-text format.

In a departure from study 1, we administered within-subject measures to assess how change in the size and anonymity of the donation would affect the acceptability of the donation (*less acceptable*, e*qually acceptable*, and *more acceptable*).

Similarly to study 1, we asked a series of questions on aspects that we believe may have influenced responses to the donation dilemma. Depending on the condition, participants were asked if they had been victims of investment fraud, data privacy or environmental violations, or racism (yes, no, and prefer not to answer), and which individuals or companies came to mind when thinking of those engaged in fraudulent investment schemes or poor consumer data privacy/environmental practices or racism. All participants were asked whether they had even been employed by the kind of institution presented in the condition and the recency of their engagement with such an institution (e.g. visit, donation, and read a newsletter). Again, we sought beliefs on the ability to maintain the anonymity of a donation (seven-point scale anchored with *extremely easy* and *extremely difficult*).

Driven by controversies in universities and beyond regarding donations from foreign government-related entities, we added a second vignette involving a foreign company partially owned by a morally tainted government. We randomized the moral taint (concerns about human rights abuses or poor environmental practices). We fixed the nature of the institution that would receive the donation with that randomly assigned in the first vignette, the size of the donation ($100,000), and that it would be publicized. Here is an example of the vignette in the human rights condition:

Consider a large firm which is partially owned by a foreign government. A {institution} has just been approached by the firm which is looking to make a donation. The government is considered by some to be a role-model in its region of the world. For others, the activities of the government raise significant concerns regarding human rights abuses. The firm is seeking to donate $100,000. This is considered by the {institution} to be a relatively large donation. The donation would be made public (i.e. the donor's name and donation amount would appear on an annual donor list).

Participants were then asked whether the institution should accept the donation, using the same six-point Likert scale from the first vignette.

Finally, basic demographic information was collected (gender identity, age, household income, education, and political preference). We dropped religiosity as it had no predictive power in study 1.

### Studies 1 and 2: demographic representativeness

We examined (i) to what extent our sample was representative in terms of gender and age—variables available through Prolific—and (ii) how any deviations from demographic representativeness as reported by Prolific from the US Census Bureau affected our results.

In study 1, we found near perfect gender representativeness. Regarding age, our sample under-represented older people (i.e. those aged 48 and over). See Table S13 for details. Our results were robust when age was included as a control variable. Unlike gender, age was associated with the likelihood of accepting a donation, but the estimates were negligible in size in absolute terms (*marginal effects* ∼ 0) and also relative to the size of the main variables of interest (see Table S8).

In study 2, participants identifying as male and, again, older participants (i.e. those aged 48 years and over) were under-represented. See Table S13 for details. When controlling for age and gender, we found no influence on the size or significance of our main variables of interest. In contrast to study 1, we found that age was not a significant control variable, whereas gender was. Specifically, men were more willing to accept donations in this study, which focused on morally ambiguous donors, and the influence of gender was smaller than that of our main variables of interest (see Table S9).

### Study 3: experimental design

Study 3 mimicked study 2, but was executed with US fundraising professionals who were members of the AFPs. One of the key differences in study 3 was that participants considered the institution they worked for (rather than a randomized institution), so that vignettes would be more realistic and familiar. The intention was to make the scenarios more realistic and avoid participants having to consider other institutions that they may not be familiar with. We also added a social norms measure tapping participant expectations of their peers’ response to the main donation dilemma. This enabled us to assess the extent to which social desirability bias may affect participants’ response to the dilemma.

The CEO and Chairman of AFP emailed members inviting them to participate in the study, which was launched in March 2021. In total, *n* = 694 participants completed the study. No compensation was offered for participation, though participants who completed the survey were eligible to win 1 of 10 prizes, each worth USD100 in the form of a charitable donation to their nominated charity. The prizes were funded by the Max Planck Institute for Human Development. While the survey was of equivalent length to that used in study 2, participants took substantially longer to complete it: an average of 12 min (IQR = 7–18 min). The survey was approved by the Internal Review Board of the Max Planck Institute for Human Development. No participants were excluded from data analysis, as per the preregistration. The final sample consisted of 442 participants identifying as female, 141 identifying as male, 2 participants who identified as nonbinary, 13 who preferred not to specify, and 96 participants who did not indicate gender; the average age was 49 years (range: 18–99 years, SD = 13.2).

The first key difference in study 3 was that the framing of the dilemmas was amended to have the fundraising professionals reflect on their own place of work, rather than a randomly assigned institution.

Here is an example of a vignette (in the condition in which a large, anonymous donation was offered by an individual representing a company thought to be involved in poor data protection practices):

Imagine that the institution where you work has just been approached by a potential donor looking to make a donation. The potential donor is a CEO from a large company. The company produces goods and services that are considered by some to change society for the better. For others, the activities of the company raise significant concerns regarding consumer data privacy. The company has no recent convictions. The potential donor is seeking to donate $100,000. This is considered by the institution to be a relatively large donation. The donation would be anonymous (i.e. the donor's name and donation amount would not appear on an annual donor list).

Participants were then asked “What should the institution do regarding this potential donation?” and selected a response using the same six-point Likert scale anchored at *definitely reject* and *definitely accept*.

In the second major difference from study 2, we elicited normative expectations of what their professional peers would do in the same situation:

Please take a moment to think about your fund-raising professional peers at the Association of Fundraising Professionals (AFP).

Of 10 AFP members participating in this survey, how many do you think would have accepted this donation?

Please select your answer carefully. If you choose the correct answer you will be entered into a random draw. This draw will have 10 winners. If you are one of the 10 winners, we will donate $100 on your behalf to your nominated charity.

Participants then chose a number from 0 to 10.

The survey then went on in the same manner to study 2 and there were only minor differences in content. We excluded the question about the last engagement with or donation to an institution, given that the professionals worked in the relevant institutions. We excluded the question about the definition of a charity, as it was not relevant in this population. We again included a question about religiosity given the uncertainty over whether it may have relevance in this domain, noting that religious institutions account for the largest share of charitable donations in the United States, followed by education in a distant second place (1). Finally, we collected demographic information on participants’ fundraising profession: years of experience, type of institution they currently work for or previously worked for (education, arts and culture, human services, religion, and other), funds raised annually (seven brackets as per an AFP internal survey plus *prefer not to answer*), professional certification (AFP's Certified Fundraising Executive, AFP's Advanced Certified Fundraising Executive, other, and none).

### Study 4: experimental design

Study 4 examined whether there were any confounds arising from differences between criminal and morally ambiguous donor vignettes. In particular, we sought to understand whether the absence of social information (i.e. that some people believed that the company changed society for the better) in the criminal condition affected participants’ judgments about the moral acceptability of donations. Similarly, we sought to understand if making the donor explicitly responsible for generating moral ambiguity (rather than indirectly responsible as the company's CEO) affected judgments in noncriminal conditions. Finally, we wanted to test whether participants still preferred morally ambiguous donors over criminal donors when we controlled for both social information and individual responsibility. The absence of confounds would support confidence in the results from studies 1–3.

Participants completed a survey posted on Prolific for a flat payment of GBP0.85 (∼USD1.05) for an average of 3 min of their time (IQR = 1.8–3.4 min) in March 2023. Inclusion criteria were being a US citizen, fluency in English (self-assessed), and a minimum approval rate of 80% in earlier studies completed on the platform. Participants from previous studies were excluded from this survey.

We sought a sample of *n* = 600 based on power analysis of the study 1 findings. Using existing data on the acceptability of morally ambiguous donations in the environmental practices domain (mean = 4.8, SD = 1.6), contemplating an effect size of 0.5 on a 6-point scale, and assuming *α* = 0.05 and power = 0.8, a sample size of *n* = 129 per condition is required for a one-sided test of differences in means (74). To be conservative, we rounded this up to 150 per condition. A pilot was conducted on a sample of 20 participants to identify any errors in the survey and to assess survey completion time.

The survey was approved by the Internal Review Board of Max Planck Institute for Human Development. No participants were excluded from data analysis, as per the preregistration. The final sample (*n* = 600) consisted of 281 participants identifying as female, 300 participants identifying as male, 12 participants who identified as nonbinary, 1 who identified as “other” and 6 who preferred not to specify; the average age was 37 years (range: 18–83 years, SD = 12.7). Consistent with studies 1 and 2 (for which Prolific could not provide a representative sample), we did not use a representative sample, though this does not cause a meaningful concern (46).

Participants provided informed consent and were randomly assigned to one of four vignette conditions: type 6 (criminal donor), type 6a (criminal donor with social information), type 7 (morally ambiguous donor), or type 7a (morally ambiguous donor with explicit individual responsibility). Types 6 and 7 were tightly aligned with the previously deployed vignettes.

We fixed the following elements of the vignettes to isolate the effects of any confounds:

- moral taint domain: environmental violation (which can also be criminal and as well as morally ambiguous behavior);
- size of donation: large;
- anonymity of donation: none (publicly disclosed); and
- institution: museum.

Each vignette begins with “Consider a museum which has just been approached by a potential donor looking to make a donation. The potential donor was a CEO at a large company.” and ends with “The potential donor is seeking to donate $100,000. This is considered by the museum to be a relatively large donation. The donation would be made public (i.e. the donor's name and donation amount would appear on an annual donor list).” The intervening text, randomly assigned, is listed below for each condition.

1. Criminal: modeled on prior scenario (type 6). “…Specifically, at the company, the potential donor was responsible for poor environmental practices. This led to criminal convictions for the CEO and the company…”
2. Criminal: extended to include social information (type 6a). “…Specifically, at the company, the donor was responsible for producing goods and services that are considered by some to change society for the better. For others, the activities of the company that raise significant concerns regarding poor environmental practices. These concerns led to criminal convictions for the CEO and the company…”
3. Morally ambiguous: modeled on previous scenario (type 7). “…The company produces goods and services that are considered by some to change society for the better. For others, the activities of the company raise significant concerns regarding poor environmental practices. These concerns have not led to any convictions for the CEO or the company…”
4. Morally ambiguous: extended to highlight individual responsibility (type 7a). “…Specifically, at the company, the donor is responsible for producing goods and services that are considered by some to change society for the better. For others, the activities of the company that raise significant concerns regarding poor environmental practices. These concerns have not led to any criminal convictions for the CEO or the company…”

We also asked the following questions.

1. Please think about controversial donors seeking to make donations to institutions such as universities, museums and health and human service organizations. A controversial donor refers to one that holds a criminal conviction or exhibits a behavior or attitude that may prompt an institution to be reluctant to accept their donation. Do you think the frequency of proposed and actual donations from controversial donors has risen over the past 5 years? [Yes/no]
2. In general, do you think that an institution receiving donations should have a policy that forbids the acceptance of donations from those with criminal convictions? [Yes/no]
3. Would you like to share any other thoughts on controversial donors? [Open text]

We removed all other questions from study 2 (e.g. within-subject measures), except for demographic questions.

### Study 5: survey design

Study 5 was conducted among US members of the AFPs. An email was sent from the Executive Vice President of the AFP Foundation for Philanthropy. A random selection of 800 members received the invitation. The survey was launched in March 2023 and was open for 8 days. Participants spent a median time of 2 min on the survey (IQR = 1.7–4.2). Participants did not receive payment for their participation but were eligible to win one of four $100 vouchers for a charity they selected. The prizes were funded by Max Planck Institute for Human Development.

In total, *n* = 52 participants completed the study. The survey was approved by the Internal Review Board of Max Planck Institute for Human Development. No participants were excluded from data analysis, as per the preregistration. The largest concentrations of respondents worked in institutions raising US$1–US$5 m annually (40%) and were focused on Human Services (38%), as opposed to Education, Arts and Culture, Religion, and other domains.

The following questions were asked in the survey.

1. Please think about major gifts donors and prospects in your past or current prospect pools. Have you ever had a donor or prospect you considered to be controversial or potentially controversial? That is, a donor or prospect that holds a criminal conviction or exhibits a behavior or attitude to the extent that it would prompt reluctance to accept their gift. [Yes/No].
1a. (If answered “yes” to Q1). Of these controversial donors or prospects, what was the nature of the controversy? Please tick all that apply [criminal conviction, criminal allegation, civil litigation, negative publicity regarding behavior or attitude, other]
1b. (If answered “yes” to Q1) For every 100 donors or prospects, please estimate how many would you consider to be controversial or potentially controversial to the extent that it would prompt reluctance to accept their gift? Please write your estimate (between 1 and 100) in the box below. [0–100]
2. In general, do you think the frequency of controversial donors has risen over the past 5 years? [Yes/no]
3. Does your organization have a policy for dealing with controversial donors? [Yes/no]
3a. (If yes), does the policy explicitly forbade accepting gifts from criminal donors? [Yes/no]
4. Size of organization. Approximately how much money did your organization raise in contributed gifts from all sources during the last fiscal year? [$1−$250,000, $250,001–$500,000, $500,001–$1 m, …. More than $20 m, Prefer not to answer]
5. Type of organization. Which of the following best describes the type of institution you currently work for [Education/Arts and Culture/Human Services/Religion/Other]

We focused on large (“major”) donations because small donations usually receive little to no scrutiny. We described donations as “major” rather than “large” and did not specify a dollar amount in order to accommodate discrepancies in what different organizations consider to be a large donation (e.g. >US$100,000 at MIT, >US$25,000 at Brown University).

### Statistical analysis

For studies 1–3, as per the preregistrations, we conducted ordinary least squares regression analysis. The dependent variable was the acceptability of a donation. We ran analyses for the dependent variable both in its numeric six-point form (capturing variation in the strength of feelings around acceptability of the donation) and in binary format, (reflecting a simple accept/reject decision). The former gives greater analytical clarity around the threshold for accepting or not accepting the donation. When using the binary form of the dependent variable, we ran logistic regressions. Two-sided tests were used with a significance level of 5%. For simple comparisons on the acceptability of donations between laypeople and the public, simple nonparametric Wilcoxon tests were used. Estimates of Cohen's *d* were made to support effect size analyses. Spearman's rho correlation tests were used to examine the strength of the relationship between moral emotions and donor acceptability. For study 4, we used one-sided Wilcoxon rank-sum tests, as per the preregistration. Chat GPT 4.0 assisted with wordsmithing and proofreading the final manuscript, and provided coding support for Study 4 and Study 5.

## Supplementary Material

pgad285_Supplementary_Data

## Preprint

This manuscript was posted as a preprint: https://psyarxiv.com/hjyp5/.
