# Supplementary material for: When tainted money should fund public goods: fundraising professional and public moral preferences
Source: PNAS Nexus. 2023 Sep 26;2(9):pgad285. doi: 10.1093/pnasnexus/pgad285 (PMC10531110; doi:10.1093/pnasnexus/pgad285)
Supplement: pgad285_Supplementary_Data [file pgad285_supplementary_data.pdf]

PNAS nexus

Supplementary Information for
When tainted money should fund public goods: Fundraising professional and public moral preferences

Zoe Rahwan* and Christina Leuker

Max Planck Institute for Human Development

*Corresponding author: Zoe Rahwan

Email: zrahwan@mpib-berlin.mpg.de

This PDF file includes:

Tables S1 to S13
Figures S1 to S10

Supplementary Figures and Tables

Supplementary Tables

Table S1: Donor Type and Acceptability of Donation

**Donor Type**	**Acceptability rating (Likert Scale, 1–6)[a]**	**Accept (%)[b]**
**Study 1: Public**		
Type 1: Nothing but good	5.6 [5.5, 5.7]	98 [97, 99]
Type 2: Violent crime (sexual assault), funds not from crime	2.9 [2.7, 3.1]	36 [31, 41]
Type 3: white-collar crime (investment fraud), funds not from crime	3.5 [3.3, 3.6]	52 [47, 57]
Type 4: White-collar crime (investment fraud), funds from crime	2.1 [1.9, 2.2]	18 [14, 22]
Type 5: White-collar crime (health fraud), funds from crime	2.4 [2.3, 2.5]	22 [18, 26]
**Study 2: Public**		
Type 6: White-collar crime (investment fraud)	3.5 [3.4, 3.6]	52 [49, 55]
Type 7: Morally ambiguous – Consumer data privacy practices	4.8 [4.7, 4.9]	89 [87, 92]
Type 8: Morally ambiguous – Environmental practices	4.8 [4.7, 4.9]	88 [85, 91]
Type 9: Morally ambiguous – Racism	4.3 [4.1, 4.4]	74 [70, 78]
Summary: Morally ambiguous (Types 7, 8, 9)	4.6 [4.6, 4.7]	84 [82, 86]
**Study 3: Fundraising professionals**		
Type 6: White-collar crime (investment fraud)	3.0 [2.8, 3.2]	37 [32, 42]
Type 7: Morally ambiguous – Consumer data privacy practices	4.6 [4.4, 4.9]	85 [78, 92]
Type 8: Morally ambiguous – Environmental practices	4.9 [4.7, 5.1]	91 [85, 96]
Type 9: Morally ambiguous – Racism	3.9 [3.6, 4.1]	63 [54,72]
Summary: Morally ambiguous (Types 7, 8, 9)	4.5 [4.3, 4.6]	80 [76, 84]

Note: Figures in brackets are 95% confidence intervals.

[a] 1: Definitely reject, 2: Likely reject, 3: Rather reject, 4: Rather accept, 5 Likely accept: 6: Definitely Accept.

[b]** Dichotomized Likert scale: 1–3: Reject; 4–6: Accept.

Table S2: Anonymity and Acceptability of Donation (%)

Donor Type	Public Donation	Anonymous Donation
**Study 1 - Public**		
Type 1: Nothing but good	98<br>[96, 100]	98<br>[96, 100]
Type 2: Violent crime (sexual assault), funds not from crime	28<br>[22, 35]	44<br>[37, 51]
Type 3: White-collar crime (investment fraud), funds not from crime	42<br>[35, 49]	61<br>[54, 68]
Type 4: White-collar crime (investment fraud), funds from crime	13<br>[8, 17]	23<br>[17, 29]
Type 5: White-collar crime (health fraud), funds from crime	18<br>[13, 24]	25<br>[19, 31]
**Study 2 - Public**		
Type 6: White-collar crime (investment fraud)	43<br>[39, 48]	59<br>[55, 63]
Type 7: Morally ambiguous - Consumer Data Privacy	88<br>[84, 92]	91<br>[87, 94]
Type 8: Morally ambiguous - Environment	85<br>[81, 90]	91<br>[88, 95]
Type 9: Morally ambiguous - Racism	67<br>[61, 73]	80<br>[75, 85]
Summary - Morally ambiguous (Types 7,8,9)	80<br>[77, 83]	87<br>[85, 90]
**Study 3 - Fundraising professionals**		
Type 6: White-collar crime (investment fraud)	30<br>[23, 37]	45<br>[37, 52]
Type 7: Morally ambiguous - Consumer Data Privacy	89<br>[80, 98]	81<br>[71, 92]
Type 8: Morally ambiguous - Environment	88<br>[79, 97]	93<br>[87, 100]
Type 9: Morally ambiguous - Racism	56<br>[43, 69]	71<br>[59,83]
Summary - Morally ambiguous (Types 7,8,9)	77<br>[71, 83]	82<br>[76, 88]

Note: Figures in brackets are 95% confidence intervals

Table S3: Size and Acceptability of Donation (%)

Donor Type	Small Donation	Large Donation
**Study 2 - Public**		
Type 6: White-collar crime (investment fraud)	54<br>[50, 58]	49<br>[45, 53]
Type 7: Morally ambiguous - Consumer Data Privacy	85<br>[81, 89]	94<br>[91, 97]
Type 8: Morally ambiguous - Environment	84<br>[79, 89]	93<br>[90, 96]
Type 9: Morally ambiguous - Racism	69<br>[63, 75]	78<br>[73, 83]
Summary - Morally ambiguous (Types 7,8,9)	79<br>[76, 82]	89<br>[86, 91]
**Study 3 - Fundraising professionals**		
Type 6: White-collar crime (investment fraud)	49<br>[42, 57]	25<br>[19, 31]
Type 7: Morally ambiguous - Consumer Data Privacy	89<br>[81, 98]	81<br>[71, 91]
Type 8: Morally ambiguous - Environment	92<br>[84, 99]	90<br>[82, 98]
Type 9: Morally ambiguous - Racism	73<br>[61, 85]	53<br>[40, 67]
Summary - Morally ambiguous (Types 7,8,9)	85<br>[79, 90]	75<br>[68, 82]
Total - All tainted donors	67<br>[62, 72]	50<br>[44, 55]

Note: Figures in brackets are 95% confidence intervals

Table S4: Acceptability in % by Institution Type and Acceptability of Donation

**Institution Type**	**Study 1** Public	**Study 2** Public	**Study 3** Fundraising professionals
Charity /Human services non-profit	52<br>[48, 56]	77<br>[75, 80]	60<br>[53, 66]
Museum (Arts and Culture)	44<br>[40, 48]	70<br>[67, 73]	66<br>[53, 78]
University (Education)	39<br>[35, 43]	65<br>[62, 68]	55<br>[46, 64]
Religious	-	-	55<br>[19, 90]
Other			52<br>[45, 60]

Note: Figures in brackets are 95% confidence intervals

Table S5: Morally Tainted Foreign Firms Acceptability of Donation in %

**Tainted Actor**	**Firm (affiliated with Government)**		**Individual**
**Type of Taint**	**Poor human rights practices**	**Poor environmental practices**	**Poor environmental practices**
Public (Study 2)	51<br>[48, 54]	74<br>[72, 77]	88<br>[85, 91]
Fundraising professionals (Study 3)	28<br>[23, 33]	56<br>[50, 61]	91<br>[85, 96]

Note: Figures in brackets are 95% confidence intervals. This study fixed other aspects of the donation (i.e., large size, not anonymous).

Table S6: Correlations Between Acceptability of Donation and Anger, Disgust, and Loss of Trust in the Institution Accepting the Donation

	**Anger**	**Disgust**	**Loss of Trust**
Public (Study 1)	−0.80***	−0.82***	0.32***
Public (Study 2)	−0.42***	−0.41***	0.52***
Fundraising professionals (Study 3)	−0.32***	−0.29***	0.72***

Note: *0.05, **0.001, *** < 0.001. Spearman correlation, two-sided test. Acceptability is measured using the 6-point Likert scale. In Study 1, the question posed was: "To what extent would the {institution type} accepting the donation affect public trust in the {institution type}? The scale was anchored with "extremely negatively affected" and "extremely positively affected" with the mid-point marked as "neutral." For Study 1, we excluded the control condition (*n* = 1,618) In Studies 2 and 3, to improve clarity, the phrasing of the question was amended to "How would the {institution type} accepting the donation affect public trust in the {institution type}?"

Table S7: Belief in Ability to Maintain Anonymity

	**Ability to maintain anonymity** (1–7 scale)
Public (Study 1)	3.79[a] [3.70, 3.89]
Public (Study 2)	3.54 [3.48, 3.60]
Fundraising professionals (Study 3)	3.80 [3.65, 3.96]

Note: The scale was anchored with ‘extremely easy (1) and ‘extremely difficult’ (7), marking 4 as an indifference point. Figures in brackets refer to the 95% confidence intervals. [a]This was only asked of participants in the ‘Anonymous’ condition.

Table S8: Study 1 Probit Regressions (Marginal Effects)

Dependent Variable: Probability of Accepting the Donation

	(1)	(2)	(3)	(4)	(5)
Violent crime (sexual assault), funds not from crime	-0.66*** [-0.71; -0.60]	-0.66*** [-0.72; -0.61]	-0.68*** [-0.74; -0.62]	-0.66*** [-0.72; -0.61]	-0.67*** [-0.72; -0.61]
White-collar crime (investment fraud), funds not from crime	-0.61*** [-0.68; -0.55]	-0.62*** [-0.68; -0.55]	-0.64*** [-0.72; -0.57]	-0.62*** [-0.68; -0.55]	-0.62*** [-0.69; -0.55]
White-collar crime (investment fraud), funds from crime	-0.72*** [-0.77; -0.68]	-0.72*** [-0.77; -0.68]	-0.74*** [-0.79; -0.69]	-0.73*** [-0.77; -0.68]	-0.73*** [-0.78; -0.68]
White-collar crime (health fraud), funds from crime	-0.71*** [-0.76; -0.66]	-0.71*** [-0.76; -0.67]	-0.72*** [-0.77; -0.67]	-0.72*** [-0.76; -0.67]	-0.72*** [-0.77; -0.67]
Anonymous	0.15*** [0.10; 0.20]	0.15*** [0.10; 0.20]	0.00 [-0.23; 0.23]	0.14*** [0.09; 0.19]	0.16*** [0.10; 0.21]
Museum		-0.10** [-0.16; -0.04]	-0.10** [-0.16; -0.04]	-0.11** [-0.17; -0.04]	-0.11** [-0.18; -0.04]
University		-0.18*** [-0.24; -0.12]	-0.18*** [-0.24; -0.12]	-0.19*** [-0.26; -0.12]	-0.19*** [-0.26; -0.12]
Violent crime (sexual assault), funds not from crime × Anonymous			0.16 [-0.07; 0.40]		
White-collar crime (investment fraud), funds not from crime × Anonymous			0.18 [-0.05; 0.41]		
White-collar crime (investment fraud), funds from crime × Anonymous			0.17 [-0.07; 0.41]		
White-collar crime (health fraud), funds from crime × Anonymous			0.09 [-0.16; 0.34]		
Previously Employed				-0.04 [-0.12; 0.04]	-0.03 [-0.12; 0.06]
Last engagement - Never				0.19 ** [0.06; 0.31]	0.17* [0.04; 0.30]
Last engagement - within the last 5 years				0.07 [-0.02; 0.16]	0.08 [-0.02; 0.18]
Last engagement - within the last month				0.02 [-0.08; 0.11]	0.04 [-0.06; 0.13]
Last engagement - within the last year				0.00 [-0.08; 0.09]	0.02 [-0.08; 0.11]
Age					-0.00 ** [-0.01; -0.00]
Male					0.04 [-0.01; 0.10]
Income - $47,000 - $77,999					-0.02 [-0.10; 0.06]
Income - $78,000 - $127,000					-0.04 [-0.12; 0.04]
Income - Less than $25,000					-0.01 [-0.10; 0.08]
Income - More than $127,000					0.02 [-0.09; 0.13]
Higher Education					-0.04 [-0.09; 0.02]
Political preference - liberal					-0.09 * [-0.17; -0.02]
Political preference - moderate					-0.05 [-0.14; 0.04]
Religion Important					-0.01 [-0.08; 0.05]
AIC	1,952	1,925	1,930	1,894	1,762
BIC	1,986	1,970	1,997	1,966	1,889
Log Likelihood	-970	-955	-953	-934	-858
Deviance	1,940	1,909	1,906	1,868	**1,716**
Number of Observations	2,019	2,019	2,019	1,985	**1,838**

Note: This table shows the marginal effects on the probability of accepting a donation from five probit models. In Model 1, we include the main variables of interest - donor type and anonymity. Model 2 extends Model 1 by including the recipient institutions. Model 3 extends model 2 by including interaction terms between the donor type and anonymity. Model 4 extends Model 2 by experimental control variables of interest - past employment and the last engagement at the relevant institution-type. Model 5 extends Model 4 by including relevant demographic variables; age, gender, income, education, political and religious preferences. Omitted factor levels: Donor Type – “Nothing but good”, Institution Type – “Charity”, Last Engagement – “More than 5 years ago”, Income – “$25,000 - $46,999, Political Preference – Moderate. ‘Being a victim’ is omitted due to multicollinearity with age and gender. The table reports marginal effects of coefficients estimated from the probit model. 95% confidence intervals of the estimates are presented in brackets. ***p<0.001, **p<0.01, *p<0.05

Table S9: Study 2 Probit Regressions (Marginal Effects)

Dependent Variable: Probability of Accepting the Donation

	(1)	(2)	(3)	(4)	(5)
Morally Ambiguous - Data Privacy	0.30*** [0.27, 0.33]				
Morally Ambiguous - Environment	0.29*** [0.26, 0.32]				
Morally Ambiguous - Racism	0.17*** [0.14, 0.21]				
Criminal		-0.33*** [-0.36, -0.29]	-0.26*** [-0.32, -0.19]	-0.26*** [-0.33, -0.19]	-0.26*** [-0.33, -0.18]
Large Donation		0.04* [0.01, 0.08]	0.18*** [0.11, 0.25]	0.19*** [0.12, 0.26]	0.19*** [0.12, 0.26]
Anonymous		0.12*** [0.08, 0.15]	0.15*** [0.08, 0.21]	0.14*** [0.07, 0.21]	0.14*** [0.07, 0.21]
Criminal × Large Donation			-0.22*** [-0.33, -0.10]	-0.23*** [-0.35, -0.11]	-0.24*** [-0.36, -0.12]
Criminal × Anonymous			0.00 [-0.09, 0.10]	0.02 [-0.08, 0.12]	0.01 [-0.09, 0.11]
Large Donation × Anonymous			-0.12* [-0.24, -0.01]	-0.13* [-0.25, -0.01]	-0.13* [-0.25, -0.01]
Criminal × Large Donation × Anonymous			0.08 [-0.05, 0.20]	0.07 [-0.06, 0.21]	0.10 [-0.03, 0.22]
Social Services Non-Profit				0.05* [0.00, 0.10]	0.05* [0.01, 0.10]
University				-0.08** [-0.14, -0.03]	-0.09*** [-0.14, -0.04]
Victim of Transgression				-0.05* [-0.09, -0.01]	-0.05* [-0.10, -0.01]
Previously Employed				-0.00 [-0.06, 0.06]	-0.01 [-0.07, 0.06]
Last engagement - Within the last year				0.03 [-0.02, 0.08]	0.01 [-0.05, 0.06]
Last engagement - within the last 5 years				0.01 [-0.05, 0.07]	-0.00 [-0.06, 0.06]
Last engagement - More than 5 years ago				0.04 [-0.02, 0.10]	0.03 [-0.04,0.09]
Last engagement - Never				0.02 [-0.06, -0.10]	0.03 [-0.05,0.11]
Age					0.00 [-0.00, 0.00]
Male					0.05** [0.02, 0.09]
Income – $25,000 - $46,999					-0.03 [-0.09, 0.03]
Income - $47,000 - $77,999					-0.04 [-0.10, 0.02]
Income - $78,000 -$127,000					-0.00 [-0.07, 0.06]
Income - More than $127,000					0.01 [-0.06, 0.08]
Higher Education					0.05* [0.01, 0.09]
Political Preference - Liberal					-0.06* [-0.11, -0.01]
Political Preference - Moderate					-0.07* [-0.14, -0.00]
AIC	2777.48	2784.07	2765.73	2586.54	2470.86
BIC	2800.89	2807.47	2812.53	2679.25	2614.86
Log Likelihood	-1384.74	-1388.03	-1374.87	-1277.27	-1210.43
Deviance	2769.48	2776.07	2749.73	2554.54	2420.86
Number of Observations	2566	2566	2566	2427	2345

Note: This table shows the marginal effects on the probability of accepting a donation from five probit models run on data from the U.S. public. In Model 1, we include the three types of morally ambiguous donors. Model 2 includes the three main variables of interest - donor type (criminal/morally ambiguous), anonymity (public/anonymous) and donation size (small/large). Model 3 extends model 2 by including interaction terms between the donor type, size of donation and anonymity. Model 4 extends Model 2 by including the institution type and experimental control variables of interest - past employment,the last engagement at the relevant institution-type and whether or not the participant was a victim of the relevant moral transgression in the vignette. Model 5 extended Model 4 by including relevant demographic variables; age, gender, income, education and political preference. Omitted factor levels: (1) Donor Type – "White-collar criminal", (2,3,4,5): Institution Type – "Museum", Last Engagement – "In the last month", Income - "Less than $25,000", Political Preference – "Conservative". The table reports marginal effects. 95% confidence intervals of the estimates are presented in brackets.. ***p<0.001, **p<0.01, *p<0.05

Table S10: Study 3 Probit Regressions (Marginal Effects)

Dependent Variable: Probability of Accepting the Donation

	(1)	(2)	(3)	(4)	(5)
Morally ambiguous - data privacy	0.40***<br>(0.34, 0.46)				
Morally ambiguous - environment	0.45***<br>(0.39, 0.50)				
Morally ambiguous - racism	0.23***<br>(0.15, 0.31)				
Criminal		-0.44***<br>(-0.51, -0.37)	-0.46***<br>(-0.59, -0.33)	-0.43***<br>(-0.59, -0.27)	-0.40***<br>(-0.59, -0.22)
Large donation		-0.20***<br>(-0.28, -0.13)	-0.19*<br>(-0.35, -0.03)	-0.21*<br>(-0.40, -0.02)	-0.22*<br>(-0.43, -0.02)
Anonymous		0.12**<br>(0.04, 0.19)	0.00<br>(-0.18, 0.18)	0.02<br>(-0.18, 0.23)	-0.00<br>(-0.22, 0.21)
Criminal × Large donation			-0.05<br>(-0.28, 0.18)	-0.01<br>(-0.28, 0.26)	-0.09<br>(-0.39, 0.21)
Criminal × Anonymous			0.17<br>(-0.04, 0.38)	0.18<br>(-0.06, 0.41)	0.21<br>(-0.04, 0.47)
Large donation × Anonymous			0.11<br>(-0.11, 0.34)	0.16<br>(-0.09, 0.41)	0.21<br>(-0.05, 0.48)
Criminal × Large donation × Anonymous			-0.15<br>(-0.48, 0.17)	-0.30<br>(-0.65, 0.06)	-0.34<br>(-0.72, 0.04)
Institution - Education				0.00<br>(-0.12, 0.13)	0.00<br>(-0.13, 0.14)
Institution - Arts				0.08<br>(-0.07, 0.24)	0.11<br>(-0.06, 0.27)
Institution - Religion				-0.25<br>(-0.55, 0.06)	-0.15<br>(-0.52, 0.22)
Institution - Other				0.03<br>(-0.08, 0.15)	0.07<br>(-0.06, 0.19)
Professional Certification - CFRE				0.07<br>(-0.30, 0.44)	0.06<br>(-0.34, 0.47)
Professional Certification - None				0.12<br>(-0.27, 0.50)	0.15<br>(-0.26, 0.57)
Professional Certification - Other				-0.03<br>(-0.45, 0.40)	-0.05<br>(-0.53, 0.43)
Funds raised : $250,001 - $500,000				-0.29<br>(-0.49, -0.09)**	-0.25<br>(-0.47, -0.02)*
Funds raised : $500,001- $1,000,000				-0.22<br>(-0.42, -0.01)*	-0.24<br>(-0.48, 0.01)
Funds raised : $1,000,001 - $5,000,000				-0.11<br>(-0.28, 0.06)	-0.09<br>(-0.28, 0.11)
Funds raised : $5,000,001 - $10,000,000				-0.08<br>(-0.30, 0.13)	-0.08<br>(-0.31, 0.15)
Funds raised : $10,000,001 - $20,000,000				-0.19<br>(-0.41, 0.04)	-0.21<br>(-0.49, 0.06)
Funds raised : More than $20,000,000				-0.10<br>(-0.32, 0.12)	-0.07<br>(-0.32, 0.18)
Victim					0.12*<br>(0.01, 0.22)
Age					-0.00<br>(-0.01, 0.00)
Male					0.12<br>(-0.01, 0.25)
Income : More than $127,000					-0.05<br>(-0.21, 0.10)
Income : $25,000 - $77,999					0.07<br>(-0.05, 0.18)
Income : $78,000 - $127,000					0.15<br>(-0.11, 0.41)
Higher education					-0.24**<br>(-0.42, -0.07)
Political preference - liberal					-0.06<br>(-0.20, 0.08)
Political preference - moderate					-0.03<br>(-0.19, 0.13)
Religiosity					0.01<br>(-0.01, 0.04)
AIC	788.49	782.63	785.91	621.85	528.04
BIC	806.66	800.8	822.25	711.58	655.15
Log Likelihood	-390.24	-387.32	-384.95	-289.93	-233.02
Deviance	780.49	774.63	769.91	579.85	466.04
Number of Observations	694	694	694	530	446

Note: This table shows the marginal effects on the probability of accepting a donation from five probit models run on data from U.S. fundraising professionals. In Model 1, we include the three types of morally ambiguous donors. Model 2 includes the three main variables of interest - donor type (criminal/morally ambiguous), anonymity (public/anonymous) and donation size (small/large). Model 3 extends model 2 by including interaction terms between the donor type, size of donation and anonymity. Model 4 extends Model 3 by including the institutionally relevant variables - the domain and size (based on annual funds raised) of the charitable institution where the participant worked and their industry specific professional qualifications. Model 5 extended Model 4 by including relevant demographic variables; age, gender, income, education, political preference and whether they were a victim of a moral transgression in the relevant vignette. For Model 1, the omitted factor level was: White-collar criminal. For models (2), (3), (4), (5), the

omitted factor levels are: Institution - Human Services, Funds raised - USD1 -USD250,000, Income - Less than $25,000. Political preference - Conservative. 95% confidence intervals of the estimates are presented in brackets. ***p<0.001, **p<0.01, *p<0.05

Table S11: Studies 2 & 3 Probit Regressions (Marginal Effects)

Dependent Variable: Probability of Accepting the Donation

	(1)	(2)	(3)
Morally ambiguous - data privacy	0.32*** (0.29, 0.34)		
Morally ambiguous - environment	0.32*** (0.29, 0.35)		
Morally ambiguous - racism	0.19*** (0.15, 0.22)		
Member of Public (vs professional fundraiser)	0.10*** (0.06, 0.14)	0.10*** (0.06, 0.14)	-0.07 (-0.15, 0.01)
Criminal (vs morally ambiguous)		-0.35*** (-0.38, -0.32)	-0.41*** (-0.48, -0.35)
Large Donation		-0.01 (-0.04, 0.03)	-0.18*** (-0.25, -0.11)
Anonymous		0.12*** (0.08, 0.15)	0.10** (0.03, 0.18)
Criminal × Member of Public			0.08* (0.00, 0.16)
Large Donation × Member of Public			0.22*** (0.15, 0.29)
Anonymous × Member of Public			0.02 (-0.06, 0.10)
AIC	3567.03	3595.54	3566.7
BIC	3597.48	3625.99	3615.41
Log Likelihood	-1778.52	-1792.77	-1775.35
Deviance	3557.03	3585.54	3550.7
Number of Observations	3260	3260	3260

Note: This table shows the marginal effects on the probability of accepting a donation from three probit models run on data from U.S. fundraising professionals and the U.S. public. In Model 1, we include the four types of donor (the omitted variable is 'White-collar Criminal') and the nature of the participant - either drawn from the public or the Association of Fundraising Professionals. In Model 2, we simplify the donor type to a binary variable (criminal/morally ambiguous) and introduce the other main variables of interest, anonymity and donation size. In Model 3, we extend Model 2 by including interaction terms between all three main variables of interest - donor type, anonymity and donation size and the participant type (Member of the Public). ***p<0.001, **p<0.01, *p<0.05

Table S12: Study 2 and Study 3 Within-Subject Measures Assessing How Change in the Size/Anonymity of a Donation Affects its Acceptability

	Less acceptable	Equally acceptable	More acceptable
**Anonymity**			
**Anonymous to Public**			
Public (Study 2)	54<br>[51, 57]	37<br>[34, 39]	9<br>[8, 11]
Fundraising professionals (Study 3)	45<br>[40, 51]	50<br>[45, 56]	4<br>[2, 7]
**Public to Anonymous**			
Public (Study 2)	11<br>[9, 12]	39<br>[37, 42]	50<br>[47, 53]
Fundraising professionals (Study 3)	13<br>[10, 18]	63<br>[58, 69]	23<br>[19, 28]
**Size**			
**Small to Large**			
Public (Study 2)	26<br>[24, 29]	40<br>[38, 43]	33<br>[31, 36]
Fundraising professionals (Study 3)	51<br>[46, 57]	46<br>[41, 52]	2<br>[1, 5]
**Large to Small**			
Public (Study 2)	24<br>[21, 26]	54<br>[51, 56]	23<br>[20, 25]
Fundraising professionals (Study 3)	7<br>[5, 11]	65<br>[59, 70]	28<br>[23, 33]

Note: Figures in brackets are 95% confidence intervals.

Table S13. Study 1 and Study 2 Age and Gender Representativeness

	Proportion (%)		
	**U.S. Census**	**Study 1**	**Study 2**
**Gender**[a]			
Male	49.0	48.6	45.8
Female	51.0	51.3	54.2
**Age (years)**[b]			
18–27	18.3	26.3	41.9
28–37	18.3	34.2	32.8
38–47	16.3	18.6	13.9
48–57	17.3	11.5	6.8
58+	29.7	9.3	4.5

[a] This excludes 1 blank entry in Study 1 and 3 ‘prefer not to say’ and 16 ‘data expired’ entries in Study 2.

[b] This excludes 36 blank answers in Study 1 and 3 nonsensical answers and 75 blank answers in Study 2.

Supplementary Figures

Figure S1. Study 1 Donation Types and Donation Acceptability

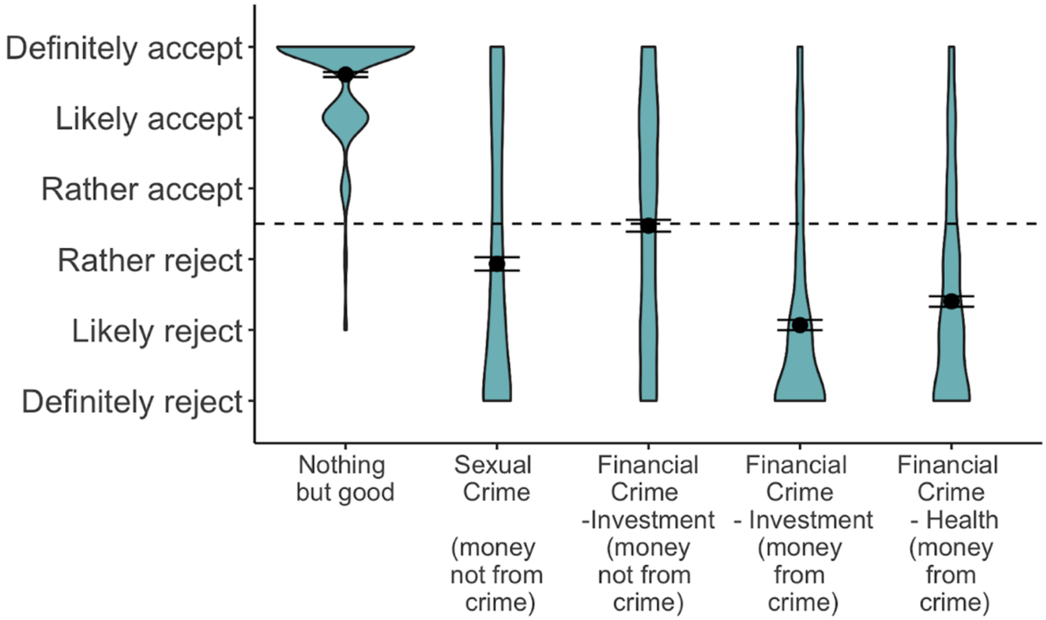

Figure S1. The distribution of individual responses regarding the permissibility of donations from different donor types presented on a 6-point Likert scale, together with mean responses and standard errors of the means. The dashed horizontal line represents the point of indifference between accepting and rejecting a donation.

Figure S2. Studies 2 and 3 Donation Types and Donation Acceptability

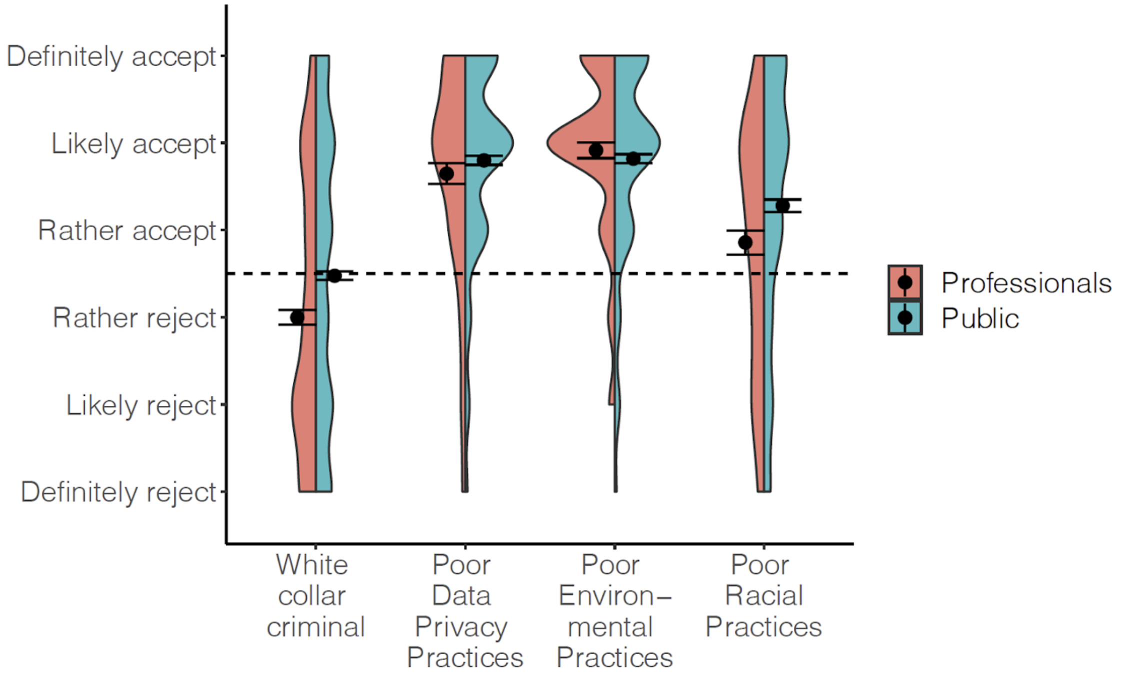

Figure S2. The distribution of individual responses from both the public and fundraising professionals regarding the permissibility of donations from different donor types, presented on a 6-point Likert scale, together with mean responses and standard errors of the means. The dashed horizontal line represents the point of indifference between accepting and rejecting a donation.

Figure S3. Study 1 Anonymity and Donation Acceptability

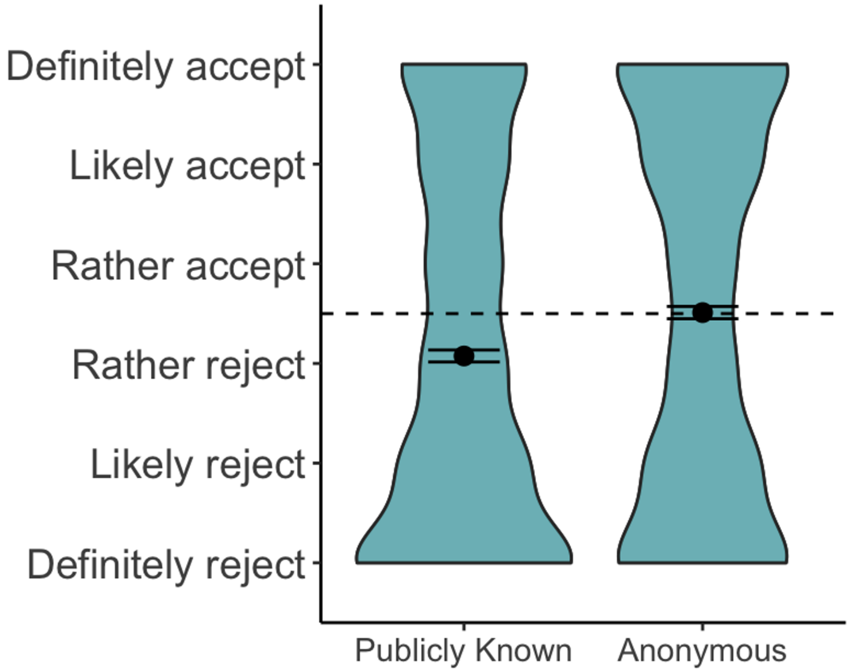

Figure S3. The distribution of individual responses regarding the permissibility of donations when either publicly known or anonymous presented on a 6-point Likert scale, together with mean responses and standard errors of the means. The dashed horizontal line represents the point of indifference between accepting and rejecting a donation.

Figure S4. Studies 2 and 3 Anonymity and Donation Acceptability

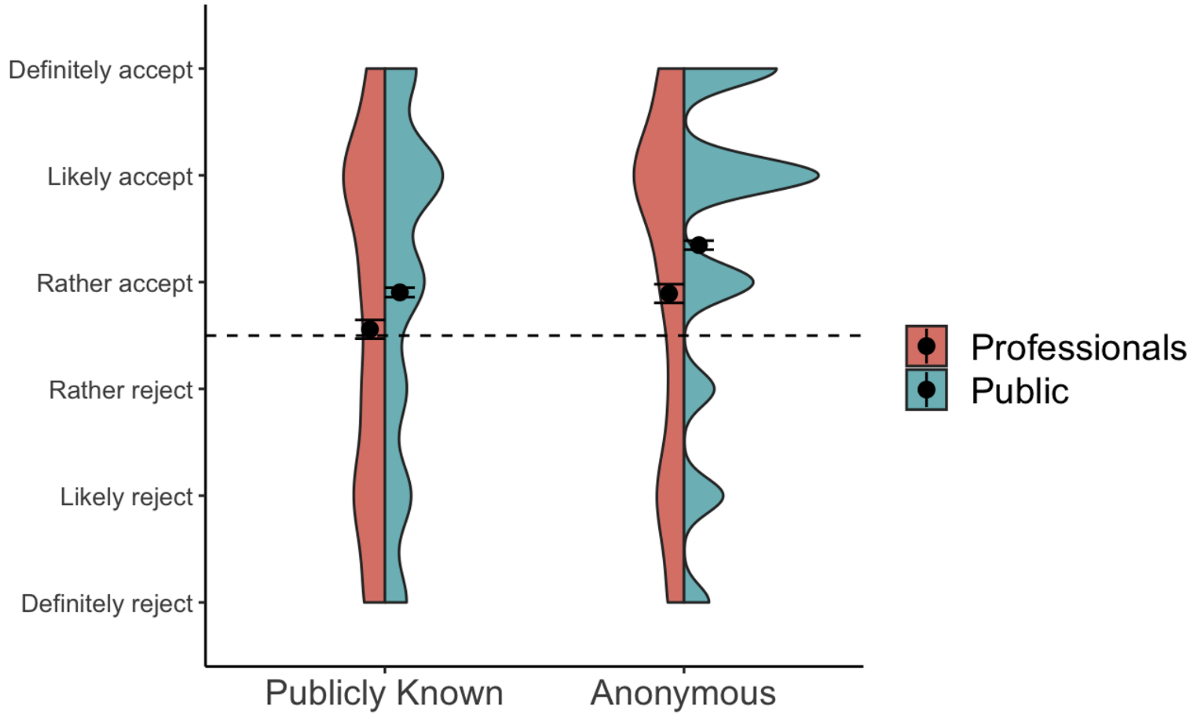

Figure S4. The distribution of individual responses from the public and fundraising professionals regarding the permissibility of donations when either publicly known or anonymous, presented on a 6-point Likert scale, together with mean responses and standard errors of the means. The dashed horizontal line represents the point of indifference between accepting and rejecting a donation.

Figure S5. Donation Size and Donation Acceptability

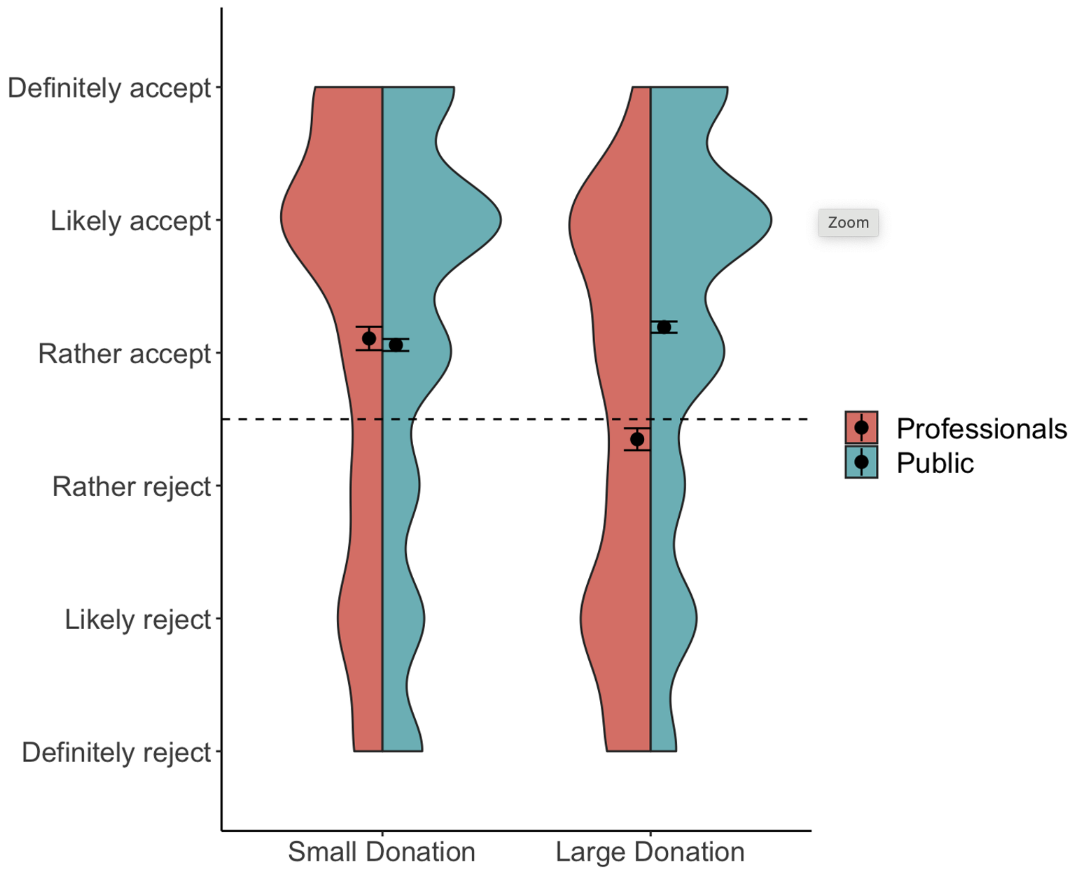

Figure S5. The distribution of individual responses from the public and fundraising professionals regarding the permissibility of donations when either small (US$100) or large (US$100,000), presented on a 6-point Likert scale, together with mean responses and standard errors of the means. The dashed horizontal line represents the point of indifference between accepting and rejecting a donation.

Figure S6. Morally Tainted Foreign Firm and Acceptability of Donation

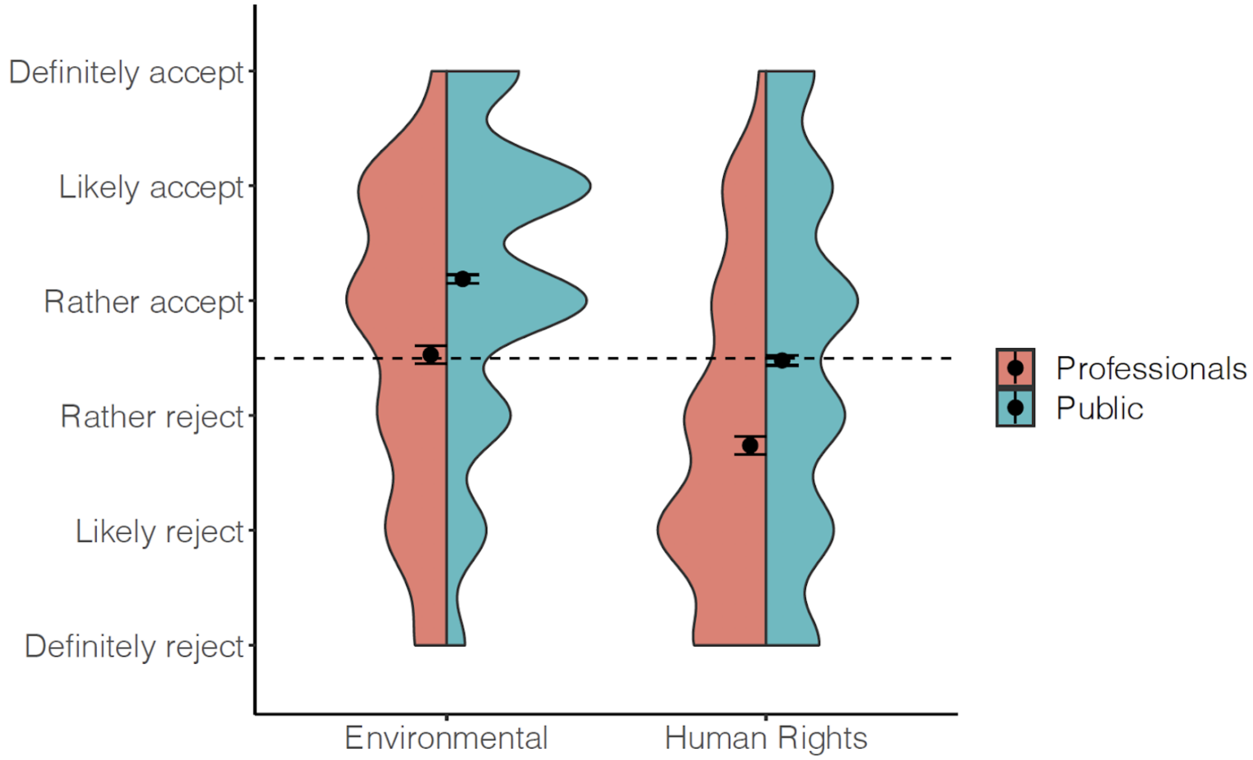

Figure S6. The distribution of individual responses from the public and fundraising professionals regarding the permissibility of donations when sourced from an entity associated with foreign government considered by some to engage in either environmental or human rights violations. The distributions are on a 6-point Likert scale, together with mean responses and standard errors of the means. The dashed horizontal line represents the point of indifference between accepting and rejecting a donation.

Figure S7. Professional Social Norms: Donor Type, Donation Size, and Anonymity

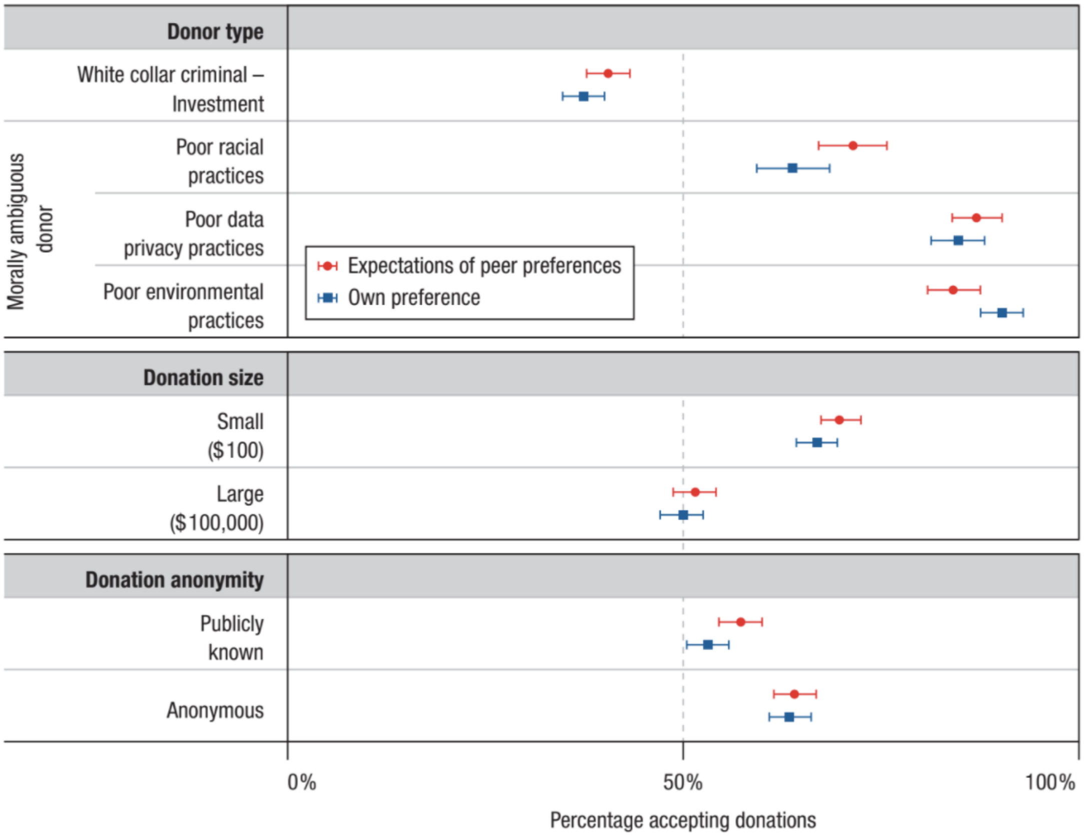

Figure S7. The acceptability of donations being proposed varied by (i) donor type, (ii) donation size, and (iii) donation anonymity according to both participants' own preferences and what they expect their peers to prefer. The figure presents the mean acceptability of donations together with standard errors of means. The dashed vertical line represents the point of indifference between accepting and rejecting a donation.

Figure S8. Acceptability of Donations by Recipient Institutions

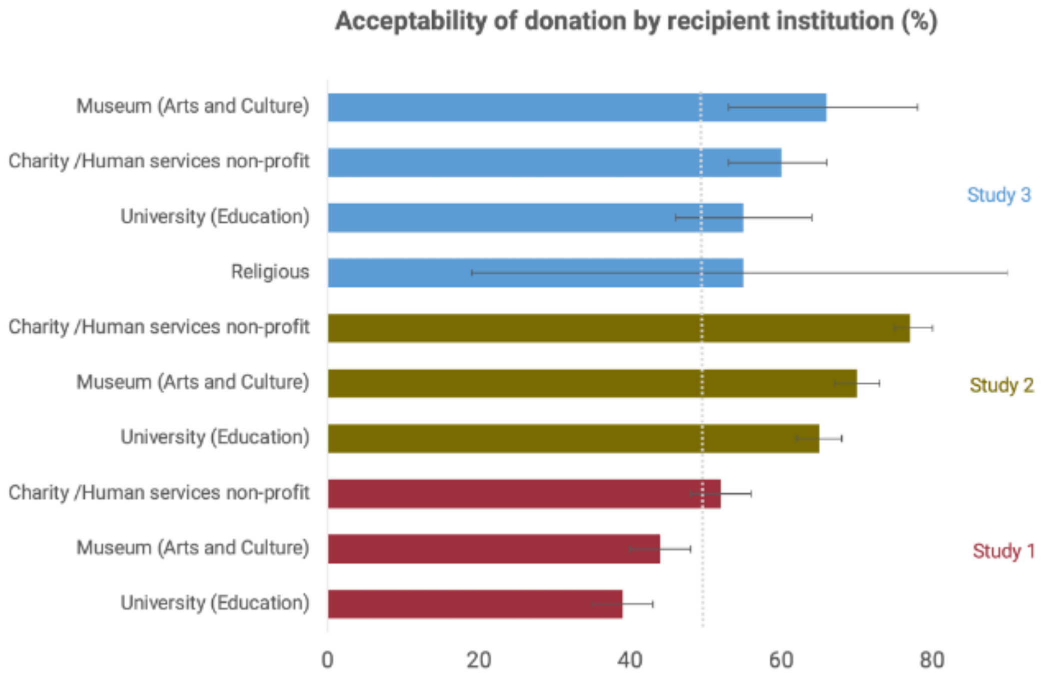

Figure S8. Donation acceptability by recipient institution. Laypeople (Studies 2 and 3; ns = 2,019, 2,566) were the most tolerant of charities receiving tainted donations relative to museums and universities. Notably, for criminal donations, those given to charities were on average acceptable, unlike those directed at museums and universities. Unlike laypeople, fundraising professionals (Study 3: n = 694) did not differentiate acceptability of tainted donations based on recipient institutions. Data are means and 95% confidence intervals.

Figure S9. Examination of Confounds

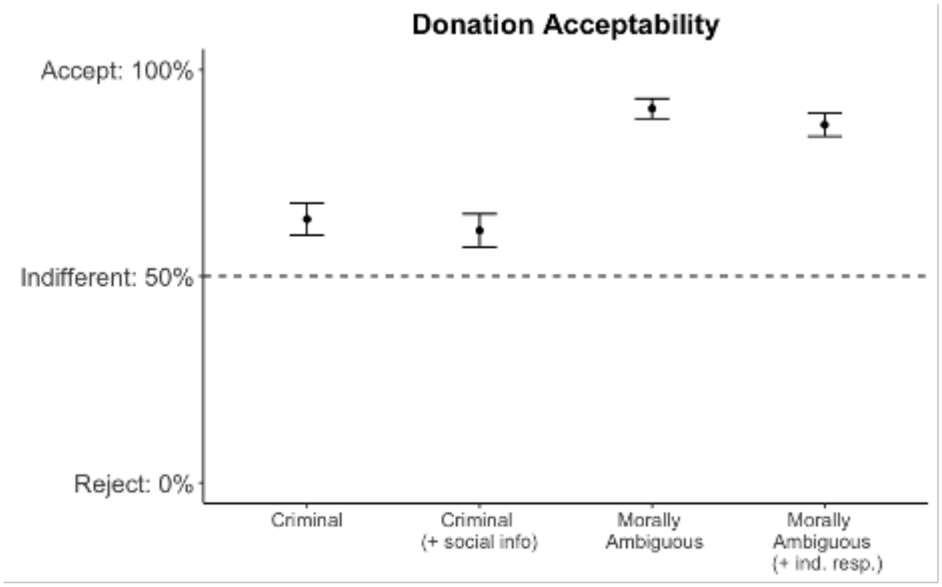

Figure S9. The acceptability of donations being proposed, varied by donor type, criminal or morally ambiguous, with and without social information and emphasis on individual responsibility in the respective vignettes. The figure presents the mean acceptability of donations to laypeople together with standard errors of means. The dashed horizontal line represents the point of indifference between accepting and rejecting a donation.

Figure S10. Donation Acceptability in Original Study 2 Condition and Re-run with Corrected Label

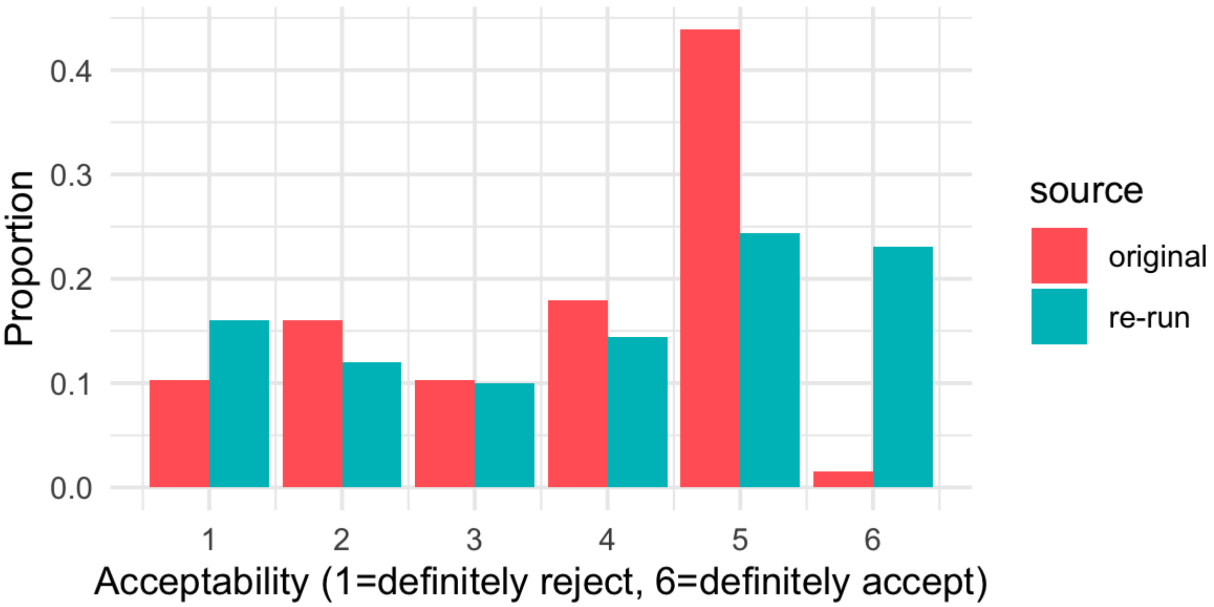

Figure S10. Proportional bar plots of the acceptability of small, anonymous donations to white collar criminals generated from original Study 2 data and the re-run study. Donation acceptability is measured on a six-point scale with each point in the scale given a text label. In the original Study 2, the far-right text label was incorrectly labelled 'definitely reject'; 'definitely accept' is the correct label. Re-running the study with the correctly labelled scale shows an increase in the proportion of respondents selecting 6 ('definitely accept'). Statistical tests indicate that there are no significant differences in either the means or the overall distributions between the data from the original study and the re-run study.
